# Harmonin homology domain-mediated interaction of RTEL1 helicase with RPA and DNA provides insights into its recruitment to DNA repair sites

**DOI:** 10.1093/nar/gkad1208

**Published:** 2023-12-28

**Authors:** Niranjan Kumar, Arushi Taneja, Meenakshi Ghosh, Ulli Rothweiler, Nagalingam Ravi Sundaresan, Mahavir Singh

**Affiliations:** Molecular Biophysics Unit, Indian Institute of Science, Bengaluru 560012, India; Department of Microbiology and Cell Biology, Indian Institute of Science, Bengaluru 560012, India; Molecular Biophysics Unit, Indian Institute of Science, Bengaluru 560012, India; The Norwegian Structural Biology Centre, Department of Chemistry, The Arctic University of Norway, N-9037, Tromsø, Norway; Department of Microbiology and Cell Biology, Indian Institute of Science, Bengaluru 560012, India; Molecular Biophysics Unit, Indian Institute of Science, Bengaluru 560012, India

## Abstract

The regulator of telomere elongation helicase 1 (RTEL1) plays roles in telomere DNA maintenance, DNA repair, and genome stability by dismantling D-loops and unwinding G-quadruplex structures. RTEL1 comprises a helicase domain, two tandem harmonin homology domains 1&2 (HHD1 and HHD2), and a Zn^2+^-binding RING domain. *In vitro* D-loop disassembly by RTEL1 is enhanced in the presence of replication protein A (RPA). However, the mechanism of RTEL1 recruitment at non-telomeric D-loops remains unknown. In this study, we have unravelled a direct physical interaction between RTEL1 and RPA. Under DNA damage conditions, we showed that RTEL1 and RPA colocalise in the cell. Coimmunoprecipitation showed that RTEL1 and RPA interact, and the deletion of HHDs of RTEL1 significantly reduced this interaction. NMR chemical shift perturbations (CSPs) showed that RPA uses its 32C domain to interact with the HHD2 of RTEL1. Interestingly, HHD2 also interacted with DNA in the *in vitro* experiments. HHD2 structure was determined using X-ray crystallography, and NMR CSPs mapping revealed that both RPA 32C and DNA competitively bind to HHD2 on an overlapping surface. These results establish novel roles of accessory HHDs in RTEL1’s functions and provide mechanistic insights into the RPA-mediated recruitment of RTEL1 to DNA repair sites.

## Graphical Abstract

**Graphical Abstract:**
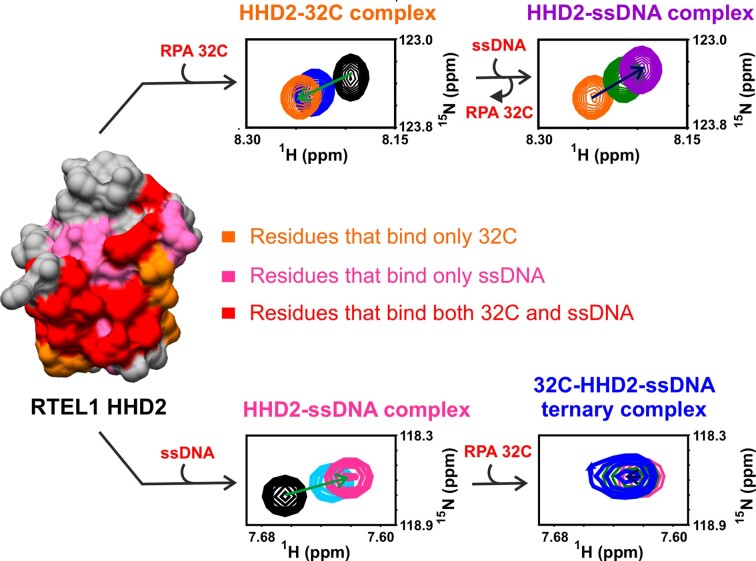

## Introduction

**Figure 1.**
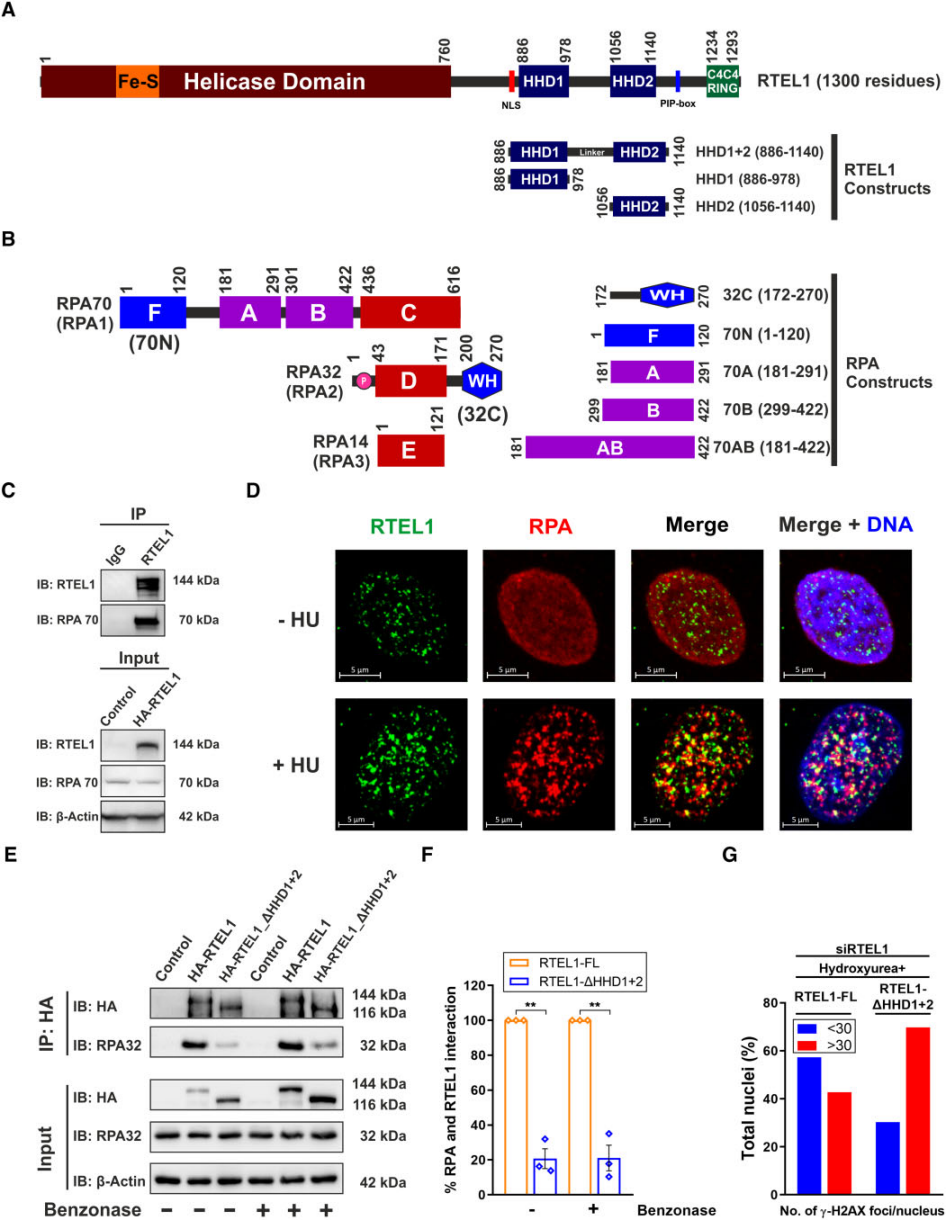
RTEL1 directly interacts with RPA. (**A**) Domain organization of human RTEL1. HHD constructs used in this work are shown with domain boundaries. (**B**) Domain organization of human RPA trimeric complex (consisting of RPA70, RPA32, and RPA14). Various constructs of RPA used in this study are shown with their domain boundaries. (**C**) Whole-cell extracts of HEK293T cells overexpressing HA-tagged human RTEL1 were immunoprecipitated (IP) with mentioned antibodies. The immunocomplexes (*upper panel*) and input (*bottom panel*) were analysed through immunoblotting (IB) with indicated antibodies. (**D**) Representative immunofluorescence images showing co-localization of endogenous RTEL1 and RPA in the nucleus of HeLa cells. Cells were either left untreated (*upper panel*) or treated (*bottom panel*) with 2 mM Hydroxyurea (HU) for 48 h, paraformaldehyde-fixed, and immuno-stained for RTEL1 (green) and RPA (red). Nuclear DNA (blue) was counterstained with Hoechst 33342. (**E**) Representative immunoblot showing co-immunoprecipitation of RPA with HA-RTEL1 and HA-RTEL1-ΔHHD1+2 in the presence or absence of Benzonase. Whole-cell extracts of HEK293T cells, overexpressing HA-tagged human RTEL1 and its deletion construct RTEL1-ΔHHD1+2, were immunoprecipitated (IP) using an anti-HA antibody. The immunocomplexes (*upper panel*) and input (*bottom panel*) were treated overnight with Benzonase and analysed through immunoblotting (IB) with indicated antibodies. (**F**) Bar graph showing % interaction between RPA and HA-RTEL1-ΔHHD1+2, relative to RPA and HA-RTEL1-FL. Data represent mean ± SEM (n = 3) using paired two-tailed Student's *t* test. Asterisks were used to represent p-values: ** *P* ≤ 0.01. (**G**) Bar graph showing % nuclei with more than or less than 30 γH2AX foci in U2OS cells (*n* = 160) overexpressing HA-RTEL1-FL and HA-RTEL1-ΔHHD1+2 under the background of siRNA mediated knockdown of endogenous RTEL1. Cells were cultured in the presence of 3 mM Hydroxyurea (HU) for 24 h and paraformaldehyde fixed. γH2AX foci were detected by immunofluorescence.

The regulator of telomere elongation helicase 1 (RTEL1) is an Fe-S cluster and DEAH motif-containing DNA helicase with ATP-dependent 5′ to 3′ helicase activity (1,2). RTEL1 plays essential roles in telomere maintenance, DNA repair, meiotic recombination, and genome-wide replication (1,3–6). It is a modular protein consisting of an N-terminal helicase domain followed by two tandem harmonin homology domains 1&2 (HHD1 and HHD2), a PCNA-interacting protein-box (PIP-box) motif, and a C-terminal C4C4 type RING domain (3) (Figure 1A). RTEL1 is an anti-recombinase and executes non-crossovers by promoting the synthesis-dependent strand annealing (SDSA) pathway of homologous recombination through disassembling displacement-loop (D-loop) intermediates during the DNA repair and meiotic recombination processes (1,7). RTEL1 interacts with the replisome's proliferating cell nuclear antigen (PCNA) through its PIP-box motif and helps in genome-wide replication (5,8). The central HHD1 and HHD2 are predicted to mediate protein-protein interactions (9). Several mutations in RTEL1 are associated with genetic diseases such as Hoyeraal-Hreidarsson syndrome (HHS) and familial pulmonary fibrosis (FPF) (3,10–13). These diseases are associated with short telomere length in patients resulting in premature aging, bone marrow failure, and predisposition to cancer.

HHDs are the most recent entrant to the newly classified αα-hub domain-containing proteins (14). The αα-hub domains typically consist of 3–5 α-helices. Hub proteins mediate fidelity in signalling and larger protein complexes. HHDs consist of five α-helices and are proposed to act mainly as a protein-protein interaction domain. So far, nine HHDs have been identified in six proteins, viz. Harmonin, Whirlin, Delphilin, PDZD7, CCM2 and RTEL1 (14–16) (Figure S1). The HHDs are associated with the PDZ domain in Harmonin, Whirlin, Delphilin, and PDZD7, with the PID domain in CCM2, and with the helicase and RING domains in RTEL1 (Figure S1), suggesting that HHDs have evolved to provide context-dependent functions in different proteins (14–16). HHD1 and HHD2 of RTEL1 harbor several mutations associated with HHS and FPF, underscoring their functional importance (9). In a recent study, a region encompassing HHD1 of RTEL1 was shown to interact with SLX4 (6,17,18). Human SLX4 is a tumor suppressor protein that has been reported to associates with structure-specific endonucleases such as ERCC, MUS81-EME, and SLX1 during interstrand DNA cross link repair, HR, and maintenance of telomere DNA (19). RTEL1–SLX4 interaction was shown to be important for preventing replication–transcription conflicts in the cell (17).

Replication protein A (RPA) is a trimeric protein complex consisting of RPA70, RPA32 and RPA14 subunits (Figure 1B) that binds single-stranded DNA (ssDNA) with high affinity in a sequence-independent manner (20). ssDNA is an intermediate product in the replication, recombination, and repair (3R) pathways in the cell (21). Since RPA and several DNA helicases (including RTEL1) are critical players in 3R pathways, they play interactive roles in maintaining genomic integrity (22). DNA helicases such as BLM, WRN and FANCJ have been shown to interact physically and functionally with RPA (10,22,23).

Homologous recombination is an essential cellular process for the accurate repair of DNA double-strand breaks (DSBs), meiotic recombination, and the restart of stalled replication forks. The D-loop is formed as an intermediate structure during homologous recombination events. The D-loop is also present within the t-loop structure of the telomere DNA (24). TRF2-mediated recruitment of RTEL1 at the telomere promotes the t-loop unwinding (5). In an *in vitro* assay, RTEL1 was shown to preferentially dissociate D-loops with 3′ invasion (25). However, the mechanism through which RTEL1 is recruited to the non-telomeric D-loop for its proper disassembly during DNA repair and meiotic recombination events remains undefined.

Here, we report that human RTEL1 directly interacts and co-localizes with RPA upon DNA damage. Interestingly, this interaction is mediated by the tandem harmonin homology domains of RTEL1. Using NMR chemical shift perturbation experiments, we show that the RTEL1–RPA interaction is mediated by the HHD2 and 32C domains of RTEL1 and RPA, respectively. We have characterized the HHDs using NMR spectroscopy and determined a 1.6 Å resolution X-ray crystal structure of HHD2 of human RTEL1. The structure of HHD2 reveals distinct positive and negative surfaces. Using NMR spectroscopy and isothermal titration calorimetry (ITC), we showed that HHD2 also interacts with DNA using the RPA 32C binding surface. This study establishes HHD2 as a novel accessory domain of RTEL1 that mediates both protein–protein and protein–DNA interactions. Interestingly, we also found that ssDNA competitively displaces the RPA 32C from the RTEL1 HHD2–RPA 32C complex. The interplay among RTEL1, RPA and DNA suggests a possible mechanism for RPA-mediated recruitment of RTEL1 at D-loops present at the DNA repair and recombination sites.

## Materials and methods

### Molecular cloning and site-directed mutagenesis

DNA sequences corresponding to HHD1 (residues 886–978) and HHD2 (residues 1056–1140) were cloned in the pET-28a (+) vector as described earlier (26) (Supplementary Table S1). The DNA sequences encoding the tandem harmonin homology domains (HHD1+2) (residues 886–1140) of human RTEL1 (Uniprot identifier Q9NZ71-6) were PCR amplified using a human RTEL1 cDNA clone as a template and subcloned into *E. coli* expression vector pET-28a (+) between NdeI and XhoI restriction endonuclease sites.

The DNA sequences encoding the 70N (residues 1–120 of RPA70), 70A (residues 181–291 of RPA70), 70B (residues 299–422 of RPA70), 70AB (residues 181–422 of RPA70) and 32C (residues 172–270 of RPA32) domains of human RPA were PCR amplified using p11d-tRPA plasmid, a gift from Marc Wold (Addgene plasmid 102613) (27) as a template and subcloned into *E. coli* expression vector pET-15b between NdeI and BamHI restriction endonuclease sites (Supplementary Tables S1 and S2).

For generating the single mis-sense mutants of HHD2, site-directed mutagenesis was performed by following the standard protocol of overlapping primer-based cloning (Supplementary Table S2). The PCR amplified products were treated with DpnI restriction enzyme before transforming into the DH5α competent cells.

The forward and reverse primer sets for each construct are listed in the Supplementary Table S2. Phusion High-Fidelity DNA polymerase (New England Biolabs; Cat. No. M0530) was used for PCR amplification of each construct. The recombinant plasmids were amplified in *E. coli* DH5α cells and isolated through the QIAprep Spin Miniprep Kit (Qiagen; Cat. No. 27106). The positive clones were confirmed through Sanger sequencing of plasmid DNA. All the cloned plasmids (Supplementary Table S1) encode a hexa-histidine (6xHis) purification tag at the N-terminus of the protein sequence except the Mammalian expression plasmids (synthesised from Genscript), which encode a HA-tagged full-length and HHD1+2 deletion constructs of RTEL1.

### Protein expression and purification

#### RTEL1 HHD1, HHD2 and HHD1+2

RTEL1 HHD1 and HHD2 domains were expressed and purified as described previously (26). In summary, the following protocol was followed for the purification of HHD1, HHD2 and HHD1+2. Plasmids containing HHD1, HHD2 and HHD1+2 were individually transformed into *E. coli* Rosetta (DE3) cells. Cells were grown in either LB (for unlabelled protein) or M9 minimal media (for uniformly ^15^N-labelled protein for NMR) in the presence of Kanamycin and Chloramphenicol antibiotics. ^15^N NH_4_Cl (Cambridge Isotope Laboratories; Cat. No. NLM-467-10) was used (1 g/l of the media) as a sole source of nitrogen in case of minimal media preparation. Protein expression was induced at OD_600_ of 0.8–1 by adding 1 mM of IPTG, and the culture was incubated at 20°C for 18 h. Cells were harvested and lysed using sonication under lysis buffer (50 mM Tris pH 8 at 4°C, 500 mM NaCl, 10% Glycerol, 0.02% NaN_3_). EDTA-free Protease Inhibitor Cocktail tablets (Roche; Cat. No. 11836170001) and 1 mM PMSF (Roche; Cat. No. 10837091001) were added before lysing the resuspended cells through sonication. The lysate was subjected to centrifugation at 13 000 rpm for 60 min at 4°C, and the supernatant was collected. 0.1% (v/v) polyethyleneimine (PEI) precipitation followed by another round of centrifugation was carried out to remove any nucleic acid contamination in the case of HHD2 and HHD1+2.

For Ni^2+^-NTA affinity chromatography, supernatant was loaded on a His Trap FF column (GE) pre-equilibrated with lysis buffer. Extensive column wash was performed with wash buffer (50 mM Tris pH 8 at 4°C, 500 mM NaCl, 20 mM Imidazole, 10% Glycerol, 0.02% NaN_3_) to remove the non-specifically bound proteins. 6xHis-tagged proteins were eluted with imidazole in the elution buffer (50 mM Tris pH 8 at 4°C, 500 mM NaCl, 250 mM imidazole, 10% glycerol, 0.02% NaN_3_).

For performing the ion-exchange chromatography, Ni^2+^-NTA eluted proteins were subjected to buffer exchange in buffer A (20 mM Tris pH 7.4 at 4°C, 50 mM NaCl, 2 mM DTT, 5% glycerol, 0.02% NaN_3_) and loaded on the ion-exchange column (HiTrap Q column for HHD1 and HiTrap Heparin column for HHD2 and HHD1+2) pre-equilibrated with buffer A. The bound protein was eluted with a linear gradient of 1M NaCl containing buffer B (20 mM Tris pH 7.4 at 4°C, 1 M NaCl, 2 mM DTT, 5% glycerol, 0.02% NaN_3_).

Finally, size-exclusion chromatography (SEC) was carried out on a Superdex 75 column (HiLoad 16/600, prep grade; GE) pre-equilibrated with the NMR buffer (20 mM Tris–HCl pH 7.4 at 25°C, 50 mM NaCl, 2 mM DTT, 0.02% NaN_3_), or crystallization buffer (20 mM Tris–HCl pH 7.4 at 25°C, 250 mM NaCl, 5% glycerol, 2 mM DTT, 0.02% NaN_3_), or ITC buffer (10 mM potassium phosphate pH 6.5 at 25°C, 50 mM KCl, 0.02% NaN_3_) depending on intended use of the protein for subsequent experiments.

SDS-PAGE analysis was performed to ascertain the purity, molecular weight (relative to Precision Plus Protein Standards Dual colour, Bio-Rad, Cat. No. 161-0394), and integrity of the purified proteins (Figure S3A). Proteins were concentrated using a centrifugal filter unit (3 kDa MWCO, Merck Millipore; Cat. No. UFC900324) at 3500 rpm and 4°C. HHD2 samples were concentrated at 15°C, owing to its lower solubility at 4°C. The concentrations of the protein were calculated by ultraviolet (UV) absorbance at 280 nm using a UV spectrophotometer (Eppendorf), and the calculated molar extinction coefficients of N-terminally hexa-histidine tagged HHD1 (ϵ = 4470 M^−1^ cm^−1^), HHD2 (ϵ = 4470 M^−1^ cm^−1^), and HHD1+2 (ϵ = 14440 M^−1^ cm^−1^) determined using EXPASY webserver (28).

#### HHD2 mutants

All single residue mutants (H1058E, R1068A, R1068E, and K1087E) of HHD2 were expressed and purified by following the above-mentioned protocol for wild type HHD2. For performing CD Spectroscopy, ^1^H 1D NMR, and ^1^H–^15^N 2D HSQC NMR titration experiments, SEC of all the mutants were carried out on a Superdex 75 column pre-equilibrated with the buffer containing 20 mM Na-Phosphate pH 7.4, 50 mM NaCl, 2 mM DTT, 0.02% NaN_3_.

SDS-PAGE analysis was performed to ascertain the purity, molecular weight, and integrity of the purified proteins. The concentrations of the protein were calculated by ultraviolet (UV) absorbance at 280 nm using a UV spectrophotometer (Eppendorf), and the calculated molar extinction coefficients of HHD2 mutants (ϵ = 4470 M^−1^ cm^−1^ for all mutants) determined using EXPASY webserver (28).

#### Full-length RPA trimeric complex

RPA complex was expressed and purified as per the protocol from the M.S. Wold lab (27,29). p11d-tRPA (123) plasmid (Supplementary Table S1) was transformed into *E. coli* BL21(DE3) strain. Cells were grown in the presence of Ampicillin antibiotics. Protein expression was induced at OD_600_ of 0.7–0.8 by adding 0.3 mM of IPTG, and the culture was incubated at 37°C for 3 h. Protein purification was performed through Affi-gel blue (Bio-Rad; Cat. No. 153-7302), Hydroxyapatite (Bio-Rad; Cat. No. 130–0420), and Q-column (GE). 1.5 M NaSCN (Sigma; Cat. No. 251410), 80 mM Potassium phosphate, and 300 mM KCl buffer were used to elute the RPA from Affi-Gel blue, Hydroxyapatite, and Q-column, respectively. Finally, dialysis of RPA was carried out in the NMR buffer containing 100 mM NaCl (20 mM Tris–HCl pH 7.4 at 25°C, 100 mM NaCl, 2 mM DTT, 0.02% NaN_3_). SDS-PAGE analysis was performed to ascertain the purity, molecular weight and integrity of the trimeric complex RPA (Figure S3B). The concentration was calculated by UV absorbance at 280 nm and using the calculated molar extinction coefficients of RPA (ϵ = 87210 M^−1^ cm^−1^ for trimeric complex) determined through EXPASY webserver (28).

#### RPA 70N, 70A, 70B, 70AB and 32C

RPA 70N, 70A, 70B, 70AB and 32C domains were over-expressed in *E. coli* BL21(DE3) cell and purified as N-terminally hexa-histidine tagged recombinant proteins, as described previously (30–33) with a few modifications. Plasmids carrying coding DNA for different RPA domains were transformed into *E. coli* BL21 (DE3) (for 70N domain) or BL21 (DE3) pLysS strains. Cells were grown in either the LB (for unlabelled protein) or M9 minimal media (for uniformly ^15^N labelled protein for NMR) in the presence of Ampicillin / Ampicillin and Chloramphenicol antibiotics. ^15^N NH_4_Cl (1 g/l of the media) was used as a sole source of nitrogen in case of minimal media preparation. Protein expression was induced at OD_600_ of 0.8–1 by adding 1 mM of IPTG, and the culture was incubated at 37°C for 4 h (70N incubated at 20°C for 18 h). The first step of purification involved His-tag–Ni^2+^-NTA affinity chromatography for all the domains. In the next step, RPA 70A, 70B and 70AB were purified using cation exchange (Heparin column). RPA 32C was purified using anion exchange (Q column). All the domains of RPA were purified using SEC on a Superdex 75 column at the final step of purification and eluted in the NMR buffer (20 mM Tris–HCl pH 7.4 at 25°C, 50 mM NaCl, 2 mM DTT, 0.02% NaN_3_).

SDS-PAGE analysis was performed to ascertain the purity, molecular weight (relative to protein standard), and integrity of the purified proteins (Figure S3D). Proteins were concentrated using a centrifugal filter unit (3 kDa MWCO, Merck Millipore) at 3500 rpm and 4°C. The concentrations of the protein were calculated by ultraviolet (UV) absorbance at 280 nm using a UV spectrophotometer (Eppendorf), and the calculated molar extinction coefficients of N-terminally hexa-histidine tagged RPA 70N (ϵ = 2980 M^−1^ cm^−1^), 70A (ϵ = 16960 M^−1^ cm^−1^), 70B (ϵ = 15470 M^−1^ cm^−1^), 70AB (ϵ = 32430 M^−1^ cm^−1^), and 32C (ϵ = 1490 M^−1^ cm^−1^) determined using EXPASY webserver (28).

### Antibodies

Anti-RTEL1, produced in Rabbit (Sigma, Cat. No. HPA067329); Anti-RPA1, produced in Mouse (Sigma, Cat. No. SAB1406399); Anti-RPA 32 kDa subunit (9H8), produced in Mouse (Santa Cruz Biotechnology, Cat. No. sc-56770); Anti-HA tag (C29F4), produced in Rabbit (Cell Signalling Technology, Cat. No. 3724); Anti-gamma H2A.X (phospho S139) antibody [3F2], produced in mouse (Abcam, Cat. No. ab22551); Anti-beta Actin, produced in Mouse (Abcam, Cat. No. ab8226); Anti-Rabbit IgG, HRP-conjugate (Millipore, Cat. No. 12–348); Anti-Mouse IgG, HRP-conjugate (Cell Signalling Technology, Cat. No. 7076); Anti-Rabbit IgG, Alexa Fluor™ 488-conjugate (Invitrogen, Cat. No. A-11008); Anti-Rabbit IgG, Alexa Fluor™ 594-conjugate (Invitrogen, Cat. No. SA5-10040); Anti-Mouse IgG, Alexa Fluor™ 488-conjugate (Invitrogen, Cat. No. A-11001); Anti-Mouse IgG, Alexa Fluor™ 594-conjugate (Invitrogen, Cat. No. A-11032)

### siRNA

Two siRNA targeting the 3′ UTR of RTEL1 gene and scrambled control were synthesised by Eurogentec. The sequences of siRNA are listed in the Supplementary Table S2.

### Cell culture and transfection

For immunoprecipitation experiments, HEK293T cells were grown in low glucose DMEM supplemented with 10% foetal bovine serum (FBS) and an antibiotic-antimycotic mix. Cells were maintained at 37°C and 5% CO_2_ in a humidified incubator. For transfection, cells were grown to 70–80% confluence, and the plasmids (pcDNA3.1(+)-N-HA empty vector, pcDNA3.1(+)-N-HA-RTEL1, and pcDNA3.1(+)-N-HA-RTEL1-ΔHHD1+2) (Supplementary Table S1) were transfected using Lipofectamine® 2000 reagent (Invitrogen; Cat. No. 11668019) according to the manufacturer's protocol. 48 h post-transfection, the cells were harvested.

For siRNA mediated RTEL1 knockdown experiments, U2OS cells were grown in high glucose DMEM supplemented with sodium pyruvate, 10% FBS, and an antibiotic-antimycotic mix. Cells were maintained at 37°C and 5% CO_2_ in a humidified incubator till 70–80% confluence. Cells were transfected with siRNA against RTEL1 using Lipofectamine® 3000 according to the manufacturer's protocol. 72 h post transfection the cells were harvested, and western blot was performed to confirm the knockdown of endogenous RTEL1 by using anti-RTEL1 antibody (Figure S2B and C).

For plasmid overexpression in the background of RTEL1 knockdown, 70–80% confluent U2OS cells were transfected with siRNA against endogenous RTEL1. 24 h post-transfection, these cells were transfected with plasmids (pcDNA3.1(+)-N-HA empty vector, pcDNA3.1(+)-N-HA-RTEL1 and pcDNA3.1(+)-N-HA-RTEL1-ΔHHD1+2) using Lipofectamine® 3000 reagent according to the manufacturer's protocol. 24 h post transfection of plasmid, the cells were treated with 3 mM hydroxyurea for 23 h. Finally, for recovery from the DNA damage, hydroxyurea-containing media was removed and the cells were maintained under 10% FBS-containing DMEM media for 1 h, post which, the cells were fixed with paraformaldehyde.

### Preparation of cell lysates

Cells were harvested from culture dishes using a cell scraper and lysed with ice-cold cell lysis buffer (20 mM Tris–HCl pH 7.5, 100 mM NaCl, 1.5 mM MgCl_2_, 1 mM EDTA, 1 mM EGTA, 1% Triton X-100, 2.5 mM sodium pyrophosphate, 1 mM sodium orthovanadate, 1 mM PMSF and 1× protease inhibitor cocktail). The lysates were cleared by centrifugation at 12 000 rpm for 10 min at 4°C, and the supernatant was collected in a fresh tube.

### Immunoprecipitation, electrophoresis and immunoblotting

Protein lysates were quantified using the Bradford assay, and 500–750 μg lysate was pre-cleared with Protein G agarose beads (Roche, Cat. No. 11719416001) for 1 h at 4°C with rotation at 5 rpm in a rotator. Pre-cleared lysates were incubated with 3 μg of specific antibody overnight at 4°C with rotation at 5 rpm in a rotator. For Benzonase treatment, 100 units of Benzonase (Merck, Cat. No. 70664) were added to the lysate in addition to the specific antibody.

The immunoprecipitated complexes were then captured on protein G agarose beads, eluted in 2× Laemmli sample buffer with 5% β-mercaptoethanol, boiled at 96°C for 5 min and electrophoresed on 10% SDS-PAGE gels. The proteins were then transferred onto 0.45 μm PVDF membrane (GE) by overnight wet transfer. The membrane was blocked for 1 h with 5% skimmed milk prepared in TBST (Tris-buffered saline supplemented with 0.05% Tween 20) at room temperature followed by incubation at 4°C overnight with specific primary antibody prepared in 5% BSA or skimmed milk in TBST (dilution range: 1:500 to 1:1000). Bound antibodies on the membrane were recognized by horseradish peroxidase-conjugated secondary antibodies incubated for 1 h at room temperature. Between each step, the blots were washed thrice for three minutes each, with TBST. Chemiluminescence was detected using ECL reagent (Bio-rad) and the images were acquired using a chemiluminescence imager (Bio-rad).

### Immunofluorescence microscopy

HeLa or U2OS cells were grown on sterilized glass coverslips placed inside a 12-well plate. To observe the co-localisation of endogenous RTEL1 and RPA foci in DNA damage condition, 2 mM Hydroxyurea (Sigma) was added to the 60–80% confluent cultures of HeLa cells for 48 h before harvesting. For assessing the DNA damage under overexpression of RTEL1 wild type and RTEL1-ΔHHD1+2 constructs in RTEL1 knockdown background, 3 mM Hydroxyurea (Sigma) was added to the 60–80% confluent cultures of U2OS cells for 23 h followed by 1 h recovery before fixation. Both HeLa and U2OS cells were fixed using 4% paraformaldehyde for 10–15 min at room temperature. Permeabilization was carried out with 0.2% Triton X-100 for 5 min at room temperature, followed by incubation with blocking buffer (5% BSA in PBS containing 0.1% Tween 20) for 60 min. The cells were incubated overnight at 4°C with indicated primary antibodies (prepared in the blocking buffer at dilutions ranging from 1:50–1:150) followed by incubation with species-specific Alexa Fluor-conjugated secondary antibodies for 1 h at room temperature. The nuclei were stained with Hoechst 33342 (Cayman Chemical) by incubation for 15 min at room temperature. The cells were washed twice with 1X PBS between each step. Finally, the cells were mounted on a clean glass cover slide using the Fluoromount-G Aqueous Mounting Medium, and the images were acquired using Zeiss LSM 880 and Andor Dragonfly spinning disk confocal microscope. The images were further processed and analysed with ZEN 3.5 (Blue edition) or Imaris 10.0.1.

### Crystallization and X-ray structure determination

The SEC fractions of RTEL1 HHD2 were concentrated up to ∼7 mg/ml and used in the crystallization trials. Initial screening was conducted using Crystal Screen HT (Hampton Research; Cat. No. HR2-130) in 72-well oil immersion plates. The rod-like crystals of HHD2 appeared within two days at 4°C in many conditions of Crystal Screen as well as in the native crystallization buffer (20 mM Tris–HCl pH 7.4 at 25°C, 250 mM NaCl, 5% glycerol, 2 mM DTT, 0.02% NaN_3_) of HHD2. Later, the HHD2 crystals were grown in the native buffer condition using the hanging drop vapour diffusion method.

The X-ray diffraction data sets were collected at ESRF beamline ID23-1 at Grenoble (France) using the PILATUS 6M pixel-array detector (DECTRIS Ltd, Switzerland). A high-resolution data set was collected at an energy of 12.7 keV $( {{\bf \lambda } = 0.976\,{\mathrm{{\AA}}}} )$. The crystals diffracted up to a maximum resolution of 1.49 Å. The diffraction data sets were processed using XDSAPP (34). The RTEL1 HHD2 structure was determined at 1.6 Å resolution by using the Molecular Replacement with Phaser (35), employing coordinates of the Alpha fold model of RTEL1 HHD2 (AF-Q9NZ71-F1) (36) as the search model. Coot (37), PHENIX (38), and REFMAC5 (39) were used for iterative model building and refinement. The *R*_work_ and *R*_free_ of the final model are 0.182 and 0.211, respectively. The quality of the final model was assessed using Coot (37), the Molprobity server (40), and the wwPDB validation server (41). Structural figures were generated using UCSF Chimera (42). The data statistics are presented in Table 2.

### DNA sample preparation

All DNA oligos (Supplementary Table S2) were purchased from Sigma-Aldrich in the lyophilized form. The stock solutions of DNA samples were prepared by dissolving the lyophilized oligos separately in the required amount of NMR buffer (20 mM Tris–HCl pH 7.4 at 25°C, 50 mM NaCl, 2 mM DTT, 0.02% NaN_3_), and ITC buffer (10 mM Potassium phosphate pH 6.5 at 25°C, 50 mM KCl, 0.02% NaN_3_) for performing the NMR and ITC titrations, respectively.

Samples of double-stranded DNAs (dsDNA-22 and dsDNA-24) and ss_ds junction DNAs (3′ss_dsDNA-24 and 5′ss_dsDNA-24) were made by mixing the equimolar amount of complementary oligo strands, followed by incubation at 95°C for 10 min. The samples were finally reannealed by snap cooling on ice for 1 h.

### Solution NMR spectroscopy

All NMR spectra (1D ^1^H, 2D ^1^H–^15^N HSQC, and 2D ^1^H–^15^N TROSY HSQC) were acquired at 25°C on a Bruker 700 MHz spectrometer equipped with a cryogenic probe or a room temperature probe. Uniformly ^15^N-labelled protein samples were prepared in the NMR buffer containing 20 mM Tris–HCl pH 7.4 at 25°C, 50 mM NaCl, 2 mM DTT, 0.02% NaN_3_ for most of the titration experiments. The buffer used for NMR titration experiments of trimeric RPA with HHD1+2, HHD2 mutants with 32C, and ssDNA-22 with HHD2 at pH 6.5 is mentioned in the protein purification section and/or result section. 10% D_2_O (v/v) (Cambridge Isotope Laboratories; Cat. No. DLM-4–25) was added to the sample for the spectrometer deuterium lock. Typically, for the titration experiments, protein sample concentration was kept at 150 μM (in case of cryoprobe) or 250 μM (room temperature probe).

The NMR data were processed using Bruker Topspin and analysed using the NMRFAM-SPARKY software (43).

The chemical shift perturbations (CSPs) were analysed as combined amide chemical shift changes with equation: Δδ_NH_ (ppm) = [(Δδ^1^H)^2^ + (Δδ^15^N/5)^2^]^1/2^, where the chemical shift changes in the ^1^H and ^15^N dimensions are denoted by Δδ^1^H and Δδ^15^N respectively.

### Fitting and simulating ^1^H–^15^N HSQC based NMR titration profiles using TITAN

TITAN (stands for TITration ANalysis) software was used to analyse the ^1^H–^15^N HSQC spectra obtained during each step of titration (44). TITAN numerically simulates the evolution of magnetization during a pulse sequence in the presence of chemical exchange (44). By data fitting and simulations of NMR spectra recorded during titrations, TITAN analysis routinely reports the *K*_d_, and *K*_off_ rate of the chemical exchange, which in conjunction with each other can report the *K*_on_ rates. The ^1^H–^15^N HSQC spectra of the titrations series, HHD2-32C, HHD2-ssDNA-22 and HHD2-dsDNA-22, were processed identically using TITAN. Two-state binding model was used to fit at least 15 residues of HHD2 showing perturbation in the chemical shift in these titrations. The errors in the fit parameters were determined by bootstrapping method as suggested in the TITAN manual. The reported errors are obtained as the standard deviation from the mean of 100 bootstrap replicas. The analysed parameters are presented in Table 1.

**Table 1.** Equilibrium dissociation constants (*K*_d_s) and kinetic parameter *K*_Off_derived, for interaction of RTEL1 HHD2 with RPA 32C and DNA, from NMR titration analysis by TITAN

S. No.	Experiment	Buffer pH	*K*_d_ (μM)	*K*_Off_ (s^−1^)
1.	HHD2–32C	7.4	359.69 ± 11.31	8460.59 ± 1643.30
2.	HHD2–ssDNA-22	7.4	14.61 ± 1.21	418.20 ± 29.07
3.	HHD2–dsDNA-22	7.4	19.66 ± 4.67	1140.08 ± 166.25
4.	HHD2–ssDNA-22	6.5	3.03 ± 0.64	437.00 ± 24.27

### Isothermal titration calorimetry (ITC)

We performed isothermal titration calorimetry (ITC) experiments to quantitate the binding of HHD2 and DNA. ITC experiments allows determination of thermodynamic parameters such as change in enthalpy (Δ*H*), change in entropy (Δ*S*), Gibbs free energy change (Δ*G*), equilibrium dissociation constant (*K*_d_), and stoichiometry (*n*) of interaction under a set of experimental conditions.

ITC experiments were performed using a Microcal iTC200 instrument (GE) at 25°C. The protein and DNA samples were prepared in ITC buffer (10 mM Potassium phosphate pH 6.5 at 25°C, 50 mM KCl, 0.02% NaN_3_) and thoroughly degassed before experiments. The sample cell was filled with 30 μM of RTEL1 HHD2 and titrated with 600 μM of the DNA in syringe. Fifteen injections of the titrant (2.5 μl per injection) were performed with a spacing of 3 min between each injection and stirring speed of 800 rpm. The integrated heat data was fitted for the one-site binding model using Origin software provided by the manufacturer. All the experiments are repeated for the data consistency. The thermodynamic parameters obtained from the representative experiments involving HHD2 and various DNAs are presented in Table 3. The reported errors are fitting errors obtained from the best fit data reported in the manuscript.

### CD spectroscopy

Circular dichroism (CD) spectroscopy was performed to assess the globular folding of all the HHD2 mutants. CD spectra were recorded on 15 μM protein sample in 1 cm path length cuvette (Hellma Analytics) using a JASCO J-715 spectropolarimeter. Three accumulations of the spectra were recorded for the wavelength range of 260–200 nm at 25°C with a scanning speed of 50 nm/min and a response time of 4 s. The buffer subtraction and the smoothening of the raw spectra were performed with the inbuilt software (JASCO). Finally, the millidegrees ellipticity data was normalized to mean residue molar ellipticity using the equation:


\begin{eqnarray*} {\rm \theta (deg\, cm^2dmol^{-1})} &=& {\rm ellipticity\, (mdeg)\times 10^6/pathlength\,(mm)}\\ &&\times\, {\rm [protein]\, (\mu M)\times number\, of\, peptide\, bonds}. \end{eqnarray*}

### Bioinformatic analysis

The disordered linker region between HHD1 and HHD2 domain was predicted through VSL2B (45) and IUPred3 (46) servers. Multalign (47) tool was used to perform the sequence alignment of the RTEL1 HHD2 domain from different vertebrate species.

### HADDOCK modelling

The NMR chemical shift perturbations (CSPs) data-driven docking of RTEL1 HHD2 and RPA 32C domain was performed using HADDOCK2.2 webserver with easy interface (48). Residues of HHD2 and 32C that showed perturbation above the average CSPs were selected as the active residues. These included residues A1059, V1060, S1061, A1062, Y1063, L1064, A1065, D1066, A1067, R1068, R1069, G1075, S1077, Q1078, L1079, L1080, A1081, A1082, T1084, K1087, D1090 and D1134 of HHD2; and residues E223, G224, F227, I242, E252, G253, H254, Y256, T258, V259, D260, D261, D262, H263, F264, S266, T267, D268 and A269 of 32C. Surrounding residues were considered passive residues.

HADDOCK clustered 153 structures (of the total 200 generated models) in 9 cluster(s), which represents 76% of the water-refined HADDOCK models (Supplementary Table S3). The best-docked clusters were ranked according to their HADDOCK score. The ranking of the clusters is based on the average HADDOCK score of the top 4 members of each cluster. The HADDOCK score of the water-refined model is calculated as:


\begin{eqnarray*} {\rm HADDOCK\, score}&=& 1.0\times E_{\rm vdw} + 0.2\times E_{\rm elec}\\ && +\, 1.0\times E_{\rm desol} + 0.1 \times E_{\rm air} \end{eqnarray*}

where *E*_vdw_ = intermolecular Van der Waals energy, *E*_elec_ = intermolecular electrostatic energy, *E*_desol_ = empirical desolvation energy, *E*_air_ = restrain energy.

The *Z*-score of a cluster indicates how many standard deviations from the average this cluster is. The more negative HADDOCK score and *Z*-score of a cluster indicate better quality.

### Quantification and statistical analysis

Quantification of co-immunoprecipitation and immunofluorescence data was performed through ImageJ software (49). The quantified value and number of independent experiments are mentioned in the figure legend (Figure 1F and G).

## Results

### RTEL1 physically and functionally interacts with RPA

In BioGRID database (50), RTEL1 is included in the interactome of the RPA–ssDNA complex (51). However, there is no report of direct interaction between RTEL1 and RPA. Both RTEL1 and RPA are involved in several DNA metabolic pathways in the cell. Therefore, we investigated for any possible physical and functional interaction between RTEL1 and RPA. First, the HEK293T cells were transfected with pcDNA3.1-N-HA-RTEL1 for transient overexpression of HA-tagged RTEL1. A co-immunoprecipitation experiment was performed, and the results showed that endogenous RPA co-immunoprecipitated with RTEL1, suggesting physical interaction between RPA and RTEL1 (Figure 1C). Since RPA and RTEL1 have essential roles in DNA repair pathways, we treated HeLa cells with hydroxyurea (a DNA damaging agent) and performed an immunofluorescence microscopy-based experiment. Upon DNA damage, a subset of endogenous RTEL1 and RPA foci co-localize (Figure 1D), thus indicating the functional nature of RTEL1–RPA interaction.

Analysis of the RPA interaction sites on the BLM, WRN and FANCJ helicases indicates that a motif/domain other than the helicase domain interacts with the RPA (Figure S2A) (22,52,53). Since the HHDs in RTEL1 were classified as putative protein-protein interaction domains (9,14), we hypothesised that HHDs could harbour potential RPA binding sites. To test this hypothesis, we overexpressed the HHD1+2 (HHD1-linker-HHD2) deletion construct of RTEL1 (pcDNA3.1-N-HA-RTEL1-ΔHHD1+2) in HEK293T cells. Deletion of HHD1+2 resulted in a significant reduction (about 80%) of co-immunoprecipitated RPA (Figure 1E and 1F), suggesting that, indeed, HHDs of RTEL1 are involved in interacting with RPA. The co-immunoprecipitation experiments performed in the presence of Benzonase (endonuclease that degrades both DNA and RNA) (Figure 1E and 1F) also gave the same results, suggesting that RPA-RTEL1 interaction is not mediated via nucleic acids.

In next experiment, the endogenous RTEL1 was knocked down in U2OS cells using siRNAs. Western blot analysis using anti-RTEL1 antibody showed about 30% reduced levels of endogenous RTEL1 upon siRNA treatment (Figure S2B and C). In the endogenous RTEL1 knocked down cells, the exogenous full-length (FL) RTEL1 (using pcDNA3.1-N-HA-RTEL1) and ΔHHD1+2 RTEL1 (using pcDNA3.1-N-HA-RTEL1-ΔHHD1+2) were transiently overexpressed under DNA damaged condition induced using hydroxyurea. The extent of DNA damage in the cells was assessed by counting γH2AX foci (using anti-γH2AX antibodies). γH2AX protein is an established marker of DNA damage in the cell. The results showed that there is an increase in number of γH2AX foci in the cells overexpressing ΔHHD1+2 RTEL1 as compared to the cells with overexpression of FL RTEL1 (Figure 1G). This data suggested that deletion of HHDs results in impairment of DNA damage repair by RTEL1.

To confirm if RTEL1–RPA physical interaction is mediated via HHDs of RTEL1, the ^15^N-labelled HHD1+2 was titrated with unlabelled human RPA and ^1^H-^15^N 2D TROSY-HSQC NMR spectra of HHD1+2 were recorded at each step of titration (Figure S3A–C). We observed resonance peak broadening and chemical shift perturbations (CSP) of several residues of HHD1+2, which indicate a direct interaction between HHD1+2 and RPA (Figure S3C).

### The tandem harmonin homology domains of RTEL1 are connected through an intrinsically disordered linker region

Although predicted to be consisting of the harmonin-N-like fold using the hydrophobic cluster analysis (9), HHD1 and HHD2 of the RTEL1 have not been characterized experimentally for their structures. Bioinformatic analysis of the tandem harmonin homology domains of RTEL1 indicates that a disordered linker region separates HHD1 and HHD2 (Figure S4A). We recorded the ^1^H-^15^N 2D HSQC NMR spectrum for purified ^15^N-labelled HHD1, HHD2 and HHD1+2 (Figure S3A and B). The spectra of HHD1 and HHD2 overlaid well on the spectrum of HHD1+2 (Figure S4B). The extra cross-peaks arising from the linker region clustered at the centre of the HHD1+2 spectrum, ∼8.2 ppm in the ^1^H dimension, also suggested the disordered nature of the linker.

No significant chemical shift perturbations were observed in the ^1^H–^15^N 2D HSQC spectrum of HHD2 when titrated with the unlabelled HHD1 (Figure S4C) showing that these two domains do not interact with each other. Overall, these results suggest that individual HHD1 and HHD2 are independently folded domains separated by a disordered linker region. We have recently reported near-complete backbone and sidechain resonance assignments of individual HHD1 and HHD2 (26).

### RPA interacts with HHD2 of RTEL1 through its winged-helix domain 32C

The largest subunit of the trimeric RPA, i.e. RPA70 (also known as RPA1), consists of four OB-fold domains (70N or 70F, 70A, 70B and 70C domains), RPA32 (also known as RPA2) subunit consists of OB-fold 32D and winged-helix (WH) 32C domains, whereas the RPA14 (also known as RPA3) subunit consists of OB-fold domain 14E (Figure 1B). Protein and DNA binding roles have been assigned to different domains of RPA. 70N, 70A, 70B and 32C domains of RPA interact with partner proteins (23,54). We took an NMR spectroscopy-based titration approach to map the interaction between HHDs of RTEL1 and different domains of RPA. Uniformly ^15^N-labelled samples of 70N, 70A, 70B, 70AB (tandem 70A and 70B), and 32C domains of RPA (Figure S3D) were titrated with the HHD1+2 domain of RTEL1. No significant CSPs were observed in the spectra of 70N, 70A, 70B, and 70AB (Figure S5A–D), while we observed distinct CSPs in the spectrum of 32C upon titration with HHD1+2 (Figure S6A). The reverse titrations (^15^N-labelled HHD1+2 titrated with RPA 32C) also showed distinct CSPs in the spectrum of HHD1+2 (Figure S6B).

**Figure 2.**
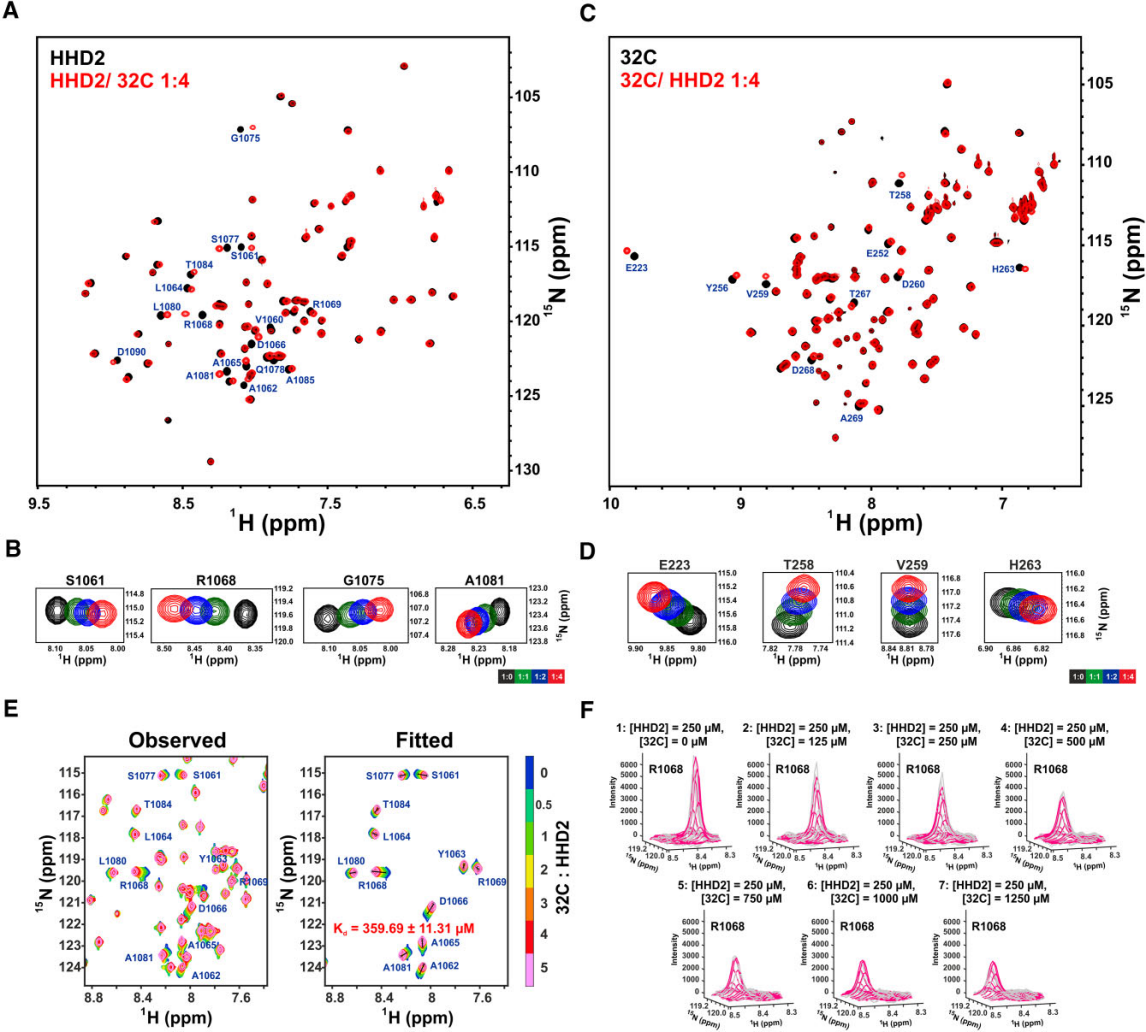
NMR titration of RTEL1 HHD2 and RPA 32C domains. (**A**) Overlay of ^1^H-^15^N HSQC spectra of ^15^N-labelled HHD2 in the absence (black) and presence (red) of RPA 32C (at 1:4 molar ratio). Residues that showed large chemical shift perturbations (CSPs) are labelled. (**B**) ^1^H-^15^N cross-peaks trajectory of representative residues S1061, R1068, G1075 and A1081 of HHD2 upon titration with RPA 32C at indicated molar ratios. (**C**) Overlay of ^1^H -^15^N HSQC spectra of ^15^N-labelled 32C in the absence (black) and presence (red) of HHD2 (at 1:4 molar ratio). Residues that showed large CSPs are labelled. (**D**) ^1^H–^15^N cross-peaks trajectory of representative residues E223, T258, V259, and H263 of 32C upon titration with HHD2 at indicated molar ratios. (**E**) 2D line shape analysis of the interaction of HHD2 with 32C using NMR TITAN software. Observed and fitted spectral regions (zoomed in) are shown with indicated molar ratio of HHD2 and 32C at each titration step. 12 residues/spins (out of total 15 spins used for the analysis) along with the Fitted K_d_ is mentioned on the spectra. (**F**) Three-dimensional overlay of observed (gray) and fitted (magenta) peak of a representative spin R1068 to show quality of the fit. Total concentration of HHD2 and 32C are mentioned at each step of titration.

In subsequent experiments, individual HHD1 and HHD2 were titrated with RPA 32C. No significant CSPs were observed in the spectrum of HHD1 (Figure S6C); however, distinct CSPs of several residues were observed in the case of HHD2 (Figures 2A and B). We also performed a reverse NMR titration of ^15^N-labelled 32C with HHD2 (Figure 2C and D). Again, we observed distinct CSPs in several residues of 32C. Interestingly, these are, mostly, same set of residues of 32C, which were perturbed upon titration with HHD1+2 (Figure S6A). Based on these results, we conclude that primarily HHD2 of RTEL1 interacts with the 32C domain of RPA. We analysed NMR CSPs observed in ^15^N-labelled HHD2 spectra upon its titrations with 32C using TITAN software (44) (please see the Materials and Methods). By data fitting and simulations of NMR spectra recorded during titrations, TITAN analysis reports the *K*_d_, and *K*_off_ rate of the chemical exchange (44). An excellent agreement between experimentally observed lineshapes and simulated lineshapes, confirm the reliability of the fit and the parameters derived from it (Figure 2E and F). This analysis reported a *K*_d_ of 359.69 ± 11.31 μM with a *K*_off_ rate of 8460.59 ± 1643.30 s^−1^ suggesting a weak interaction between HHD2 and 32C under the NMR buffer condition (Table 1).

### Structural basis of RTEL1 HHD2 and RPA 32C interaction

**Figure 3.**
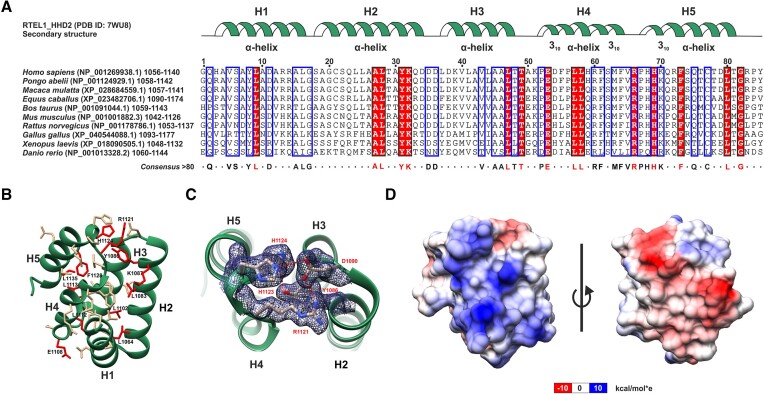
Structure of HHD2 domain of RTEL1. (**A**) Multiple sequence alignment of HHD2 of RTEL1 proteins from different vertebrate species. The secondary structure corresponding to the HHD2 of human RTEL1 is depicted at the top (green ribbon). The consensus sequence, including the conserved residues (red), is shown at the bottom. (**B**) Crystal structure of HHD2 domain of human RTEL1. Sidechains of evolutionarily conserved residues are shown as stick models (fully conserved residues and partially conserved residues are shown in red and gold, respectively). (**C**) Electron density map of a representative region consisting of conserved residues Y1086, D1090, R1121, H1123, and H1124. One salt bridge (D1090 OD1–R1121 NH1) and two H-bonds (Y1086 OH–H1124 ND1 and D1090 OD2–H1123 NE2) are shown (red dash line). (**D**) Surface electrostatic potential map of HHD2. One side (consisting of H1, H2, and H4 helices) of the protein has positively charged surface (blue), while the other side (consisting of H2, H3, and H5 helices) has negatively charged surface (red).

To understand the structural basis of RPA 32C and RTEL1 HHD2 interaction, we determined the structure of HHD2 at a resolution of 1.6 Å (Table 2) using X-ray crystallography (details in the Materials and Methods section). The structure revealed that HHD2 consists of five helices, from H1 to H5, connected by a short loop. All the helices are mainly α-helical (3.6_13_ helix) except the N- and C-termini of H4 and the N-terminal of H5, which consists of short 3_10_ helices (Figure 3A and B). The secondary structure as seen in the crystal structure of HHD2 matched well with the NMR chemical shift indexing-based secondary structure (26). The overall topology of RTEL1 HHD2 is similar to the N-terminal domain of the human Harmonin (9). A sequence alignment of HHD2 corresponding sequences of RTEL1 across the vertebrates, showed that the evolutionarily conserved residues are mainly present in the core region stabilizing the tertiary structure (Figure 3A-C). The electrostatic surface potential of HHD2 revealed a distinct pattern of positively and negatively charged opposite surfaces (Figure 3D).

**Table 2.** Crystallographic data collection and structure refinement statistics of RTEL1 HHD2

PDB ID	7WU8
**Integration**	
Space group	*P* 1 2_1_ 1
Unit cell constants	*a* = 65.64 Å, *b* = 60.96 Å, *c* = 79.71 Å
	α = 90.0º, β = 95.64º, γ = 90.0º
Wavelength (Å)	0.9763
Resolution range (Å)	48.16–1.60 (1.70–1.60)
Observed reflections	278293 (42947)
Unique reflections	80150 (12698)
Data completeness (%)	96.8 (95.3)
< I/σ(I) >	1.56 (at 1.6 Å)
R_meas_ (%)	5.3 (64.0)
CC (1/2)	99.9 (80.2)
**Refinement**	
Number of reflections	78046
*R*_work_, *R*_free_	0.182, 0.211
No. of molecules in A.S.U.	7
Total number of atoms	Total: 5128,
	Solvent: 734, non-solvent: 4394
Average *B*, all atoms (Å^2^)	30.0
R.M.S.D.	
bond length(Å) / angles (º)	0.015/ 1.895
Ramachandran outliers	
Favored/allowed/outliers (%)	100/0/0
Clash score	3
Molprobity score	1.01 (100th percentile)

Highest resolution shell is shown in parentheses.

**Figure 4.**
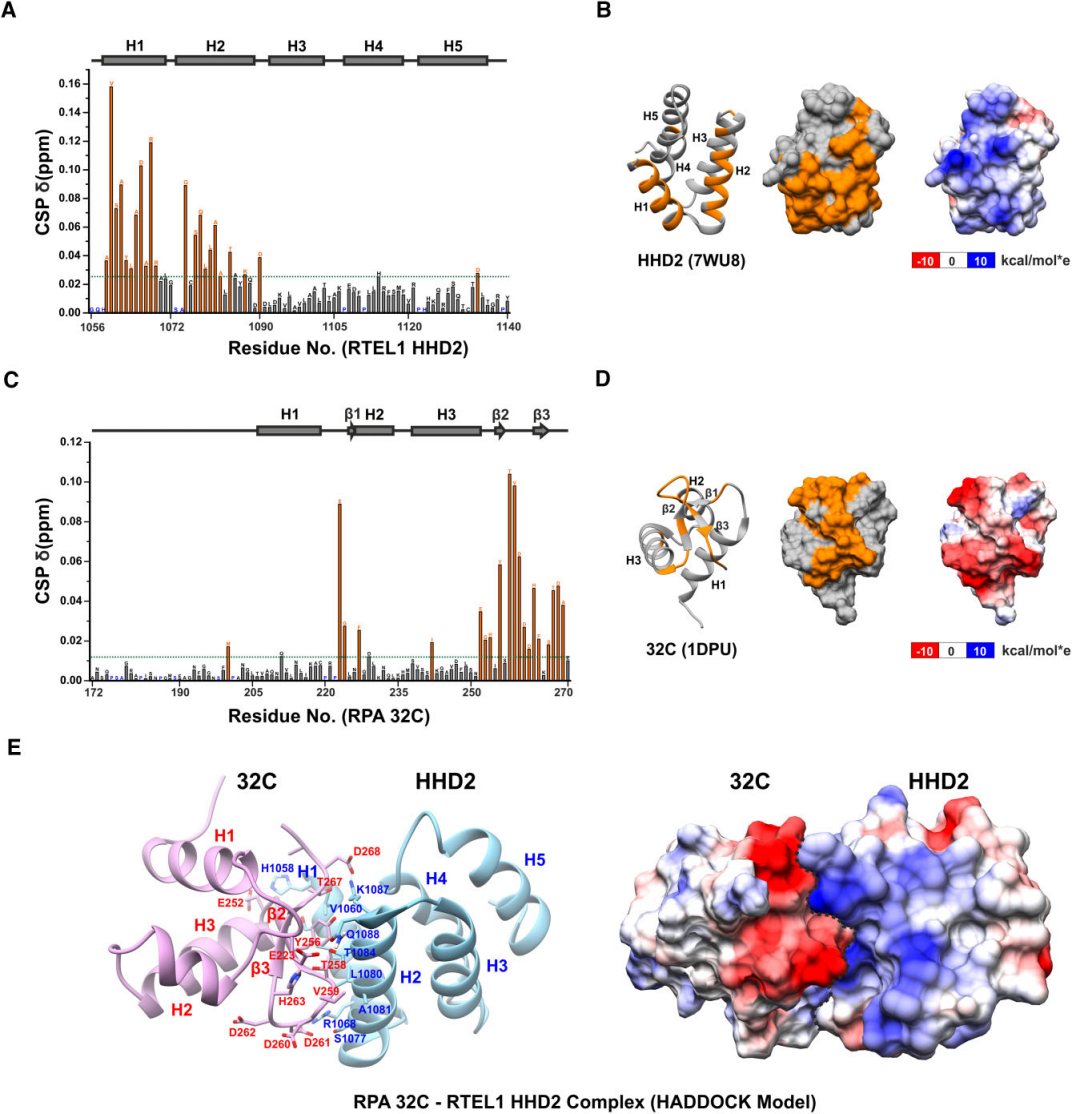
The binding interface of RTEL1-RPA interaction. (**A**) Quantitation of CSPs in HHD2 upon titration with RPA 32C (at 1:4 molar ratio). Residues with more than average CSP (green dash line) are marked as an orange bar and considered as significantly perturbed residues. Proline and unassigned residues are marked in blue. The secondary structure corresponding to HHD2 is shown at the top. (**B**) Significantly perturbed residues (orange) are marked on the structure of HHD2 (ribbon form on the *left* and surface form in the *middle*). Most of these residues lie on the positively charged surface (on the *right*) of HHD2. (**C**) Quantitation of CSPs in 32C upon titration with HHD2 (at 1:4 molar ratio). Residues with more than average CSP (green dash line) are marked as an orange bar and considered as significantly perturbed residues. Proline and unassigned residues are marked in blue. The secondary structure corresponding to 32C is shown at the top. (**D**) Significantly perturbed residues (orange) are marked on the structure of 32C (ribbon form - left and surface form - middle). Most of these residues lie on the negatively charged surface (right) of 32C. (**E**) HADDOCK model of RTEL1 HHD2–RPA 32C complex with several interface residues marked. Positively (blue) and negatively (red) charged interacting surfaces of HHD2 and 32C, respectively, are shown with dotted line (black) representing the interface.

Our recently deposited chemical shifts of HHD2 (BMRB entry 51077) (26) were used to map the RPA 32C binding surface on the HHD2 crystal structure. The observed CSPs in the titration of ^15^N-labelled HHD2 with 32C (Figure 2A) were calculated and plotted (Figure 4A). HHD2 residues (A1059, V1060, S1061, A1062, Y1063, L1064, A1065, D1066, A1067, R1068, R1069, G1075, S1077, Q1078, L1079, L1080, A1081, A1082, T1084, K1087, D1090 and D1134) that showed perturbation more than the average CSPs upon 32C binding were mainly present in the N-terminal helices H1 and H2 (Figure 4B).

Similarly, the observed CSPs in the titration of ^15^N-labelled 32C with HHD2 (Figure 2C) were calculated and plotted (Figure 4C) to map the HHD2 binding surface on the RPA 32C domain. A previously determined structure (PDB ID 1DPU) and deposited chemical shifts (BMRB entry 4460) of RPA 32C were used for the analysis (30). The residues (M200, E223, G224, F227, I242, E252, G253, H254, Y256, T258, V259, D260, D261, D262, H263, F264, S266, T267, D268 and A269) that showed perturbation more than the average CSPs upon HHD2 binding were mainly present in the C-terminal β-sheet and the adjacent loops of 32C (Figure 4D). Interestingly, this region of 32C has been shown to interact with several proteins involved in the DNA repair and replication processes (Figure S7) (55). Based on these observations, we conclude that RTEL1–RPA interaction is mediated through the conserved binding surfaces on HHD2 of RTEL1 and winged-helix domain 32C of RPA.

The electrostatic surface potential analysis, in combination with NMR CSPs, revealed that the positively charged surface of HHD2 could interacts with the negatively charged surface of 32C (Figure 4B and D). To get a molecular insight into this interaction, we generated NMR CSPs data-driven HADDOCK model of the HHD2–32C complex (Figure 4E). The largest HADDOCK cluster (cluster 1) contains 79 models out of the 153 final clustered models, with the best HADDOCK score (–78.6 ± 3.0) and *Z*-score (-1.9) (Supplementary Table S3), suggesting that the selected HADDOCK cluster is of good quality. Therefore, we chose this cluster to represent the HHD2–32C complex. The selected model satisfies the complementarity in surface charge potentials and observed NMR CSPs in 32C and HHD2. A closer inspection of the selected model showed that hydrophobic and ionic interactions stabilize the HHD2–32C complex (Figure 4E).

To validate some of the interactions as observed in the HHD2–32C HADDOCK model, we generated four point mutants of HHD2. These include mutants H1058E, R1068E/A (R1068E and R1068A), and K1087E from HHD2. These residues were found perturbed significantly in the HHD2–32C NMR titrations and reside at the interface of HHD2 and 32C in the HADDOCK model (Figure 4A and E). H1058 of HHD2 is close to E252 of 32C making an ion-pair interaction, R1068 of HHD2 interacts electrostatically with D260 and D261 of 32C, and K1087 makes a salt-bridge interaction with D268 of 32C (Figure 4E).

**Figure 5.**
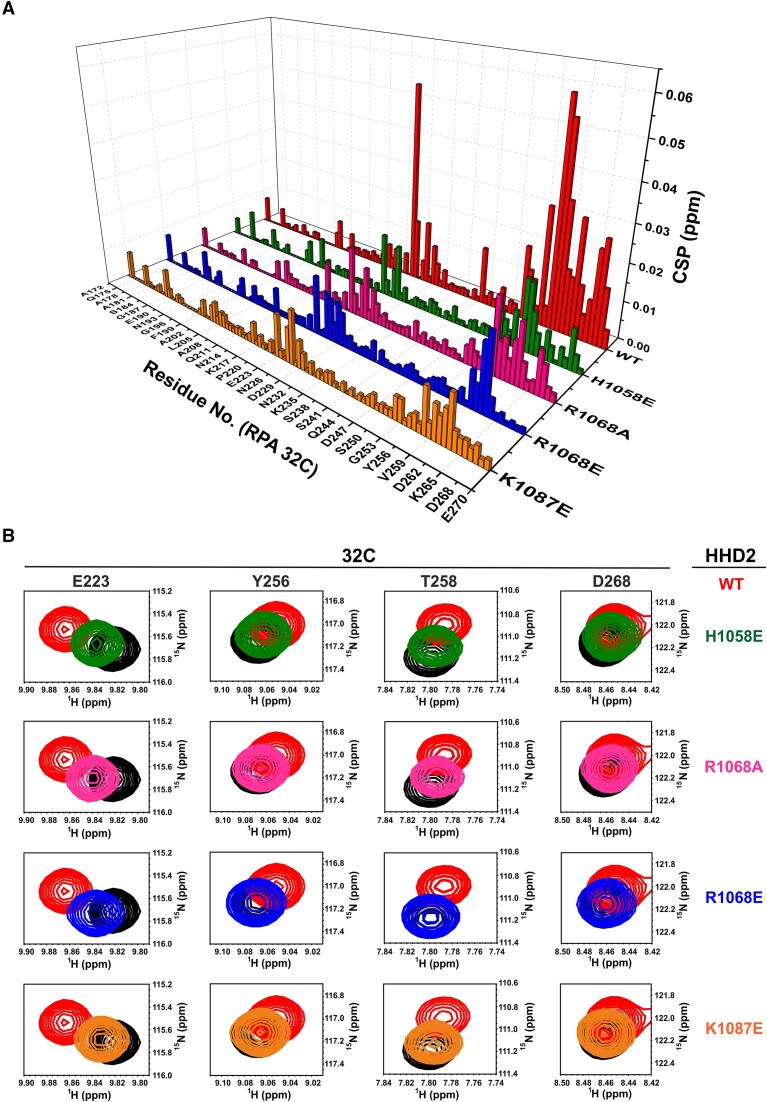
32C–HHD2 Interaction surface validation through site-directed mutagenesis of HHD2 residues. (**A**) 3D bar plot showing quantitation of CSPs in 32C upon titration with HHD2 WT (red), HHD2 H1058E (green), HHD2 R1068A (magenta), HHD2 R1068E (blue) and HHD2 K1087E (orange) (all at 1:2 molar ratio). (**B**) ^1^H–^15^N cross-peaks trajectory of representative residues (black) E223, Y256, T258 and D268 of 32C upon titration with wild type (red) and different mutants (green, magenta, blue, and orange) of HHD2 at 1:2 molar ratio. CSPs of each representative residues of 32C are shown for titration with all four mutants of HHD2. In each *inset*, CSP with wild type (WT) HHD2 is shown for comparison.

The parent residues were changed to either Alanine or oppositely charged residue to perturb the electrostatic interaction mediated by these residues, as seen in the HADDOCK model structure of HHD2–32C. HHD2 mutant proteins were expressed and purified in large scale (Figure S8A). The CD and 1D ^1^H spectra showed that all of these mutants are well folded in solution (Figure S8B and S8C). Uniformly ^15^N-labelled 32C was titrated with mutant HHD2 proteins individually (Figure S9A-E) and titration was followed by recording ^1^H–^15^N HSQC spectra at each step of titration. Compared to the WT HHD2–32C titration, we observed significantly reduced chemical shift perturbations in 32C upon its titration with HHD2 mutants (Figure 5A). The result is exemplified in Figure 5B that shows the chemical shifts of four selected residues of 32C (E223, Y256, T258, and D268) in free and in complex with the WT and mutant HHD2 proteins at 1:2 molar ratio. The extent of chemical shift perturbations in 32C residues is reduced in case of mutant HHD2 than the WT HHD2 (Figure 5B). These results suggest a weaker interaction between 32C and HHD2 mutants. Therefore, the NMR titration results suggest that the selected residues (H1058, R1068, and K1087) of HHD2 are involved in HHD2–32C binding and provide experimental support for the proposed NMR CSP driven HHD2–32C HADDOCK model structure.

### HHD2 of RTEL1 interacts with DNA

DNA helicases such as WRN and BLM contain a helicase-and-RNaseD-C-terminal (HRDC) domain, an accessory domain comprising of five α-helices, that regulate their helicase activity through binding to the DNA (2,56). We hypothesized that HHDs of RTEL1 may have similar accessory roles and can potentially interact with DNA.

**Figure 6.**
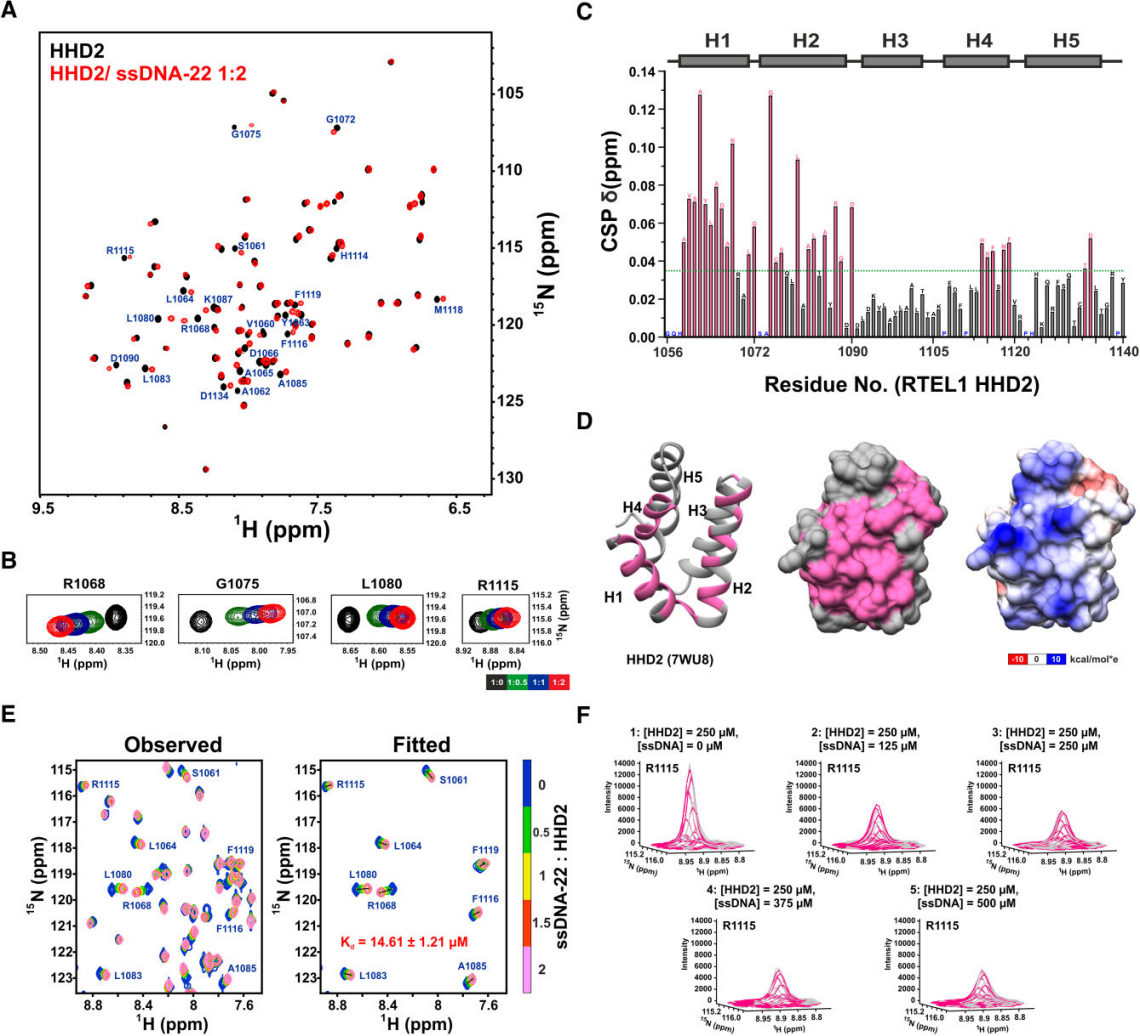
NMR titration of HHD2 and ssDNA-22. (**A**) Overlay of ^1^H–^15^N HSQC spectra of HHD2 in the absence (black) and presence (red) of ssDNA-22 (at 1:2 molar ratio). Residues that showed large CSPs are labelled. (**B**) ^1^H–^15^N cross-peaks trajectory of representative residues R1068, G1075, L1080, and R1115 of HHD2 upon titration with ssDNA-22 at indicated molar ratios. (**C**) Quantitation of CSPs in HHD2 upon titration with ssDNA-22 (at 1:2 molar ratio). Residues that showed more than average CSP (green dash line) are marked as a pink bar and considered as significantly perturbed residues. Proline and unassigned residues are marked in blue. The secondary structure of HHD2 is shown at the top. (**D**) Significantly perturbed residues (pink) are marked on the structure of HHD2 (ribbon form on the *left* and surface form in the *middle*). Most of these residues lie on the positively charged surface (on the *right*) of HHD2. (**E**) 2D line shape analysis of the interaction of HHD2 with ssDNA-22 using NMR TITAN software. Observed and fitted spectral regions (zoomed in) are shown with indicated molar ratio of HHD2 and ssDNA-22 at each titration steps. Nine residues/spins (out of total 15 spins used for the analysis) along with the Fitted *K*_d_ is mentioned on the spectra. (**F**) Three-dimensional overlay of observed (gray) and fitted (magenta) peak of a representative spin R1115 to show quality of the fit. Total concentration of HHD2 and ssDNA-22 are mentioned at each step of titration.

In the first set of experiments, we titrated HHD2 with 6-mer ssDNA (ssDNA-6), 12-mer ssDNA (ssDNA-12), and 22-mer ssDNA (ssDNA-22) (Supplementary Table S2) at physiological pH of 7.4 and followed the titrations by recording ^1^H–^15^N HSQC NMR spectra of HHD2 at each step. There were no significant CSPs observed in the 2D ^1^H–^15^N HSQC spectra of HHD2 upon titration with ssDNA-6 (Figure S10A). However, we observed distinct CSPs in the HHD2 spectra upon titration with ssDNA-12 (Figure S10B), and ssDNA-22 (Figure 6A and B). These results clearly show that the HHD2 interacts with ssDNA and the length of ssDNA should be greater than 6 nucleotides.

The extent of the observed CSPs in HHD2 was maximum in the case of ssDNA-22 titration. The observed CSPs in HHD2–ssDNA-22 titrations, were quantitated and plotted against the residues of HHD2 (Figure 6C). The HHD2 residues (A1059, V1060, S1061, A1062, Y1063, L1064, A1065, D1066, A1067, R1068, L1071, G1072, G1075, C1076, S1077, L1080, A1082, L1083, A1085, K1087, Q1088, D1090, H1114, R1115, F1116, M1118, F1119, T1133 and D1134) that showed perturbation more than the average CSPs upon ssDNA-22 binding (Figure 6C) were mapped to the helices H1, H2, and H4 of the HHD2 structure that correspond to the positively charged surface of HHD2 (Figure 6D). We analysed the NMR CSPs observed in ^15^N-labelled HHD2 upon its titrations with ssDNA-22 using TITAN software (44) (analysed similar to HHD2-32C titration) (Figure 6E). An excellent agreement between experimental line shapes and simulated line shapes were observed that confirm the reliability of the fit and the parameters derived from it (Figure 6E and F). This analysis reported a *K*_d_ of 14.61 ± 1.21 μM with a *K*_off_ rate of 418.20 ± 29.07 s^−1^ for the HHD2–ssDNA-22 interaction (Table 1).

In next set of experiments, HHD2 was titrated with dsDNA-22 at pH 7.4 and the titration was followed by recording ^1^H-^15^N HSQC spectra of HHD2 at each step. We observed distinct CSPs in the HHD2 spectra upon addition of dsDNA-22 (Figure S11A and S11B). HHD2 residues (A1059, V1060, A1062, Y1063, A1065, D1066, R1068, G1072, G1075, Q1078, L1079, L1080, A1082, L1083, A1085, K1087, Q1088, D1090, H1114, R1115, F1116, F1119, V1120, H1124, F1128, S1129, T1133, D1134, L1135 and Y1140) that showed large CSPs upon dsDNA-22 binding (Figure S11C) were mapped to the helices H1, H2, H4, and H5 of the HHD2 structure (Figure S11D). CSPs were analysed using TITAN (Figure S11E and S11F) that revealed a global *K*_d_ of 19.66 ± 4.67 μM with a *K*_off_ rate of 1140.08 ± 166.25 s^−1^ for the HHD2-dsDNA-22 interaction (Table 1). Slightly lower *K*_d_ value and approximately 2.7 times lower *K*_off_ rate (at pH 7.4) suggest that HHD2 might have preference for binding ssDNA than the dsDNA.

To check if HHD1 could also bind DNA, we performed NMR titration of ^15^N-labelled HHD1 with ssDNA-22 and dsDNA-22. Surprisingly, there were no significant CSPs observed in the 2D ^1^H-^15^N HSQC spectra of HHD1 upon ssDNA titrations (Figure S12A). In the case of dsDNA titrations, we observed minor perturbations in chemical shifts of a few residues of HHD1 (Figure S12B). These results show that HHD1 of RTEL1 either does not bind (ssDNA-22) or binds DNA very weakly (dsDNA-22).

### ITC experiments showed that HHD2 interacts with DNA of different length, sequence, and structure

To gain detailed insights into the HHD2–DNA interaction, we performed ITC experiments using DNA sequences that differ in sequence, length, and structure (single-stranded, double-stranded, and overhang containing double-stranded DNAs) (Supplementary Table S2). The ITC experiments presented here, were all performed at pH 6.5 due to the consistency of results obtained at this condition.

**Figure 7.**
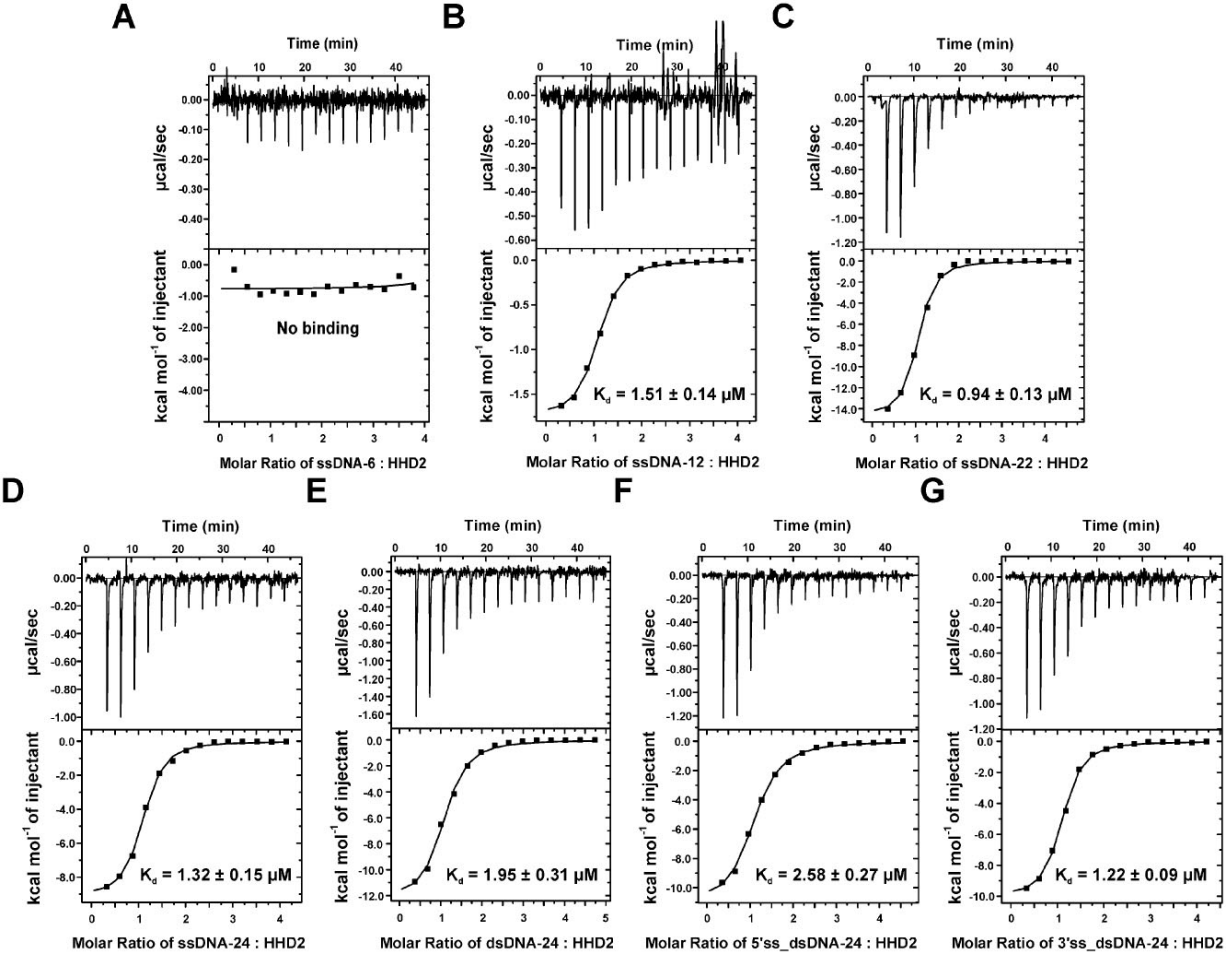
Isothermal titration calorimetry of HHD2 and DNA. The equilibrium Kd obtained upon fitting the raw data is mentioned in each panel. (**A**) Raw and fitted ITC binding isotherms for the interaction of HHD2 with ssDNA-6. No binding was observed between HHD2 and ssDNA-6. (**B**) Raw and fitted ITC binding isotherms for the interaction of HHD2 with ssDNA-12. (**C**) Raw and fitted ITC binding isotherms for the interaction of HHD2 with ssDNA-22. (**D**) Raw and fitted ITC binding isotherms for the interaction of HHD2 with ssDNA-24. (**E**) Raw and fitted ITC binding isotherms for the interaction of HHD2 with dsDNA-24. (**F**) Raw and fitted ITC binding isotherms for the interaction of HHD2 with 5′ss_dsDNA-24. (**G**) Raw and fitted ITC binding isotherms for the interaction of HHD2 with 3′ss_dsDNA-24.

We first performed the ITC experiments for HHD2 binding with 6-mer ssDNA (ssDNA-6), 12-mer ssDNA (ssDNA-12), and 22-mer ssDNA (ssDNA-22) (Supplementary Table S2). ITC titration experiment revealed that six nucleotides long ssDNA-6 sequence does not interact with HHD2 (Figure 7A). This result was in agreement with NMR titration results, which revealed no significant chemical shift perturbations in HHD2–ssDNA-6 titrations (Figure S10A). These results showed that the minimum length of DNA that can form complex with HHD2, should be greater than six nucleotides. Single stranded ssDNA-12 and ssDNA-22 sequences showed interaction with HHD2 (Figure 7B and C) with *K*_d_ values of 1.51 ± 0.14 and 0.94 ± 0.13 μM, respectively derived using ITC titrations (Table 3). NMR titration experiments had also revealed that HHD2 interacts with ssDNA-12 and ssDNA-22 (Figure S10B and Figure 6).

**Table 3.** Equilibrium dissociation constants (*K*_d_s) and other thermodynamic parameters for RTEL1 HHD2 and DNA interactions using ITC experiments at pH 6.5 *(errors are fitting errors from the reported data sets)*

S. No.	Experiment	*K*_d_ (μM)	Δ*G* (kcal/mol)	Δ*H* (kcal/mol)	*T*Δ*S* (kcal/mol)	*n*
1.	HHD2–ssDNA-6	No binding	−	−	−	−
2.	HHD2–ssDNA-12	1.51 ± 0.14	−7.93 ± 0.73	−1.76 ± 0.03	6.17 ± 0.76	1.01 ± 0.01
3.	HHD2–ssDNA-22	0.94 ± 0.13	−8.21 ± 1.16	−14.74 ± 0.30	−6.53 ± 1.46	0.95 ± 0.01
4.	HHD2–ssDNA-24	1.32 ± 0.15	−8.01 ± 0.92	−9.21 ± 0.16	−1.20 ± 1.08	1.01 ± 0.01
5.	HHD2–dsDNA-24	1.95 ± 0.31	−7.78 ± 1.25	−12.48 ± 0.41	−4.70 ± 1.66	1.00 ± 0.02
6.	HHD2–5′ss_dsDNA-24	2.58 ± 0.27	−7.61 ± 0.80	−11.3 ± 0.27	−3.69 ± 1.07	1.03 ± 0.02
7.	HHD2–3′ss_dsDNA-24	1.22 ± 0.09	−8.06 ± 0.66	−10.11 ± 0.12	−2.05 ± 0.78	1.01 ± 0.01

We have also probed interaction of HHD2 with ssDNA-22 at the lower pH of 6.5 using NMR titration to compare it with ITC titrations performed at the same pH (Figure S13A). Chemical shifts perturbations of several residues were observed. The TITAN analysis of the CSPs (Figure S13B) reported a *K*_d_ of 3.03 ± 0.64 μM compared to *K*_d_ of 0.94 ± 0.13 μM derived using ITC method at pH 6.5, which are lower than the *K*_d_ of 14.61 ± 1.21 μM at pH 7.4 (Tables 1 and 3). The isoelectric point of HHD2 is 8.73. Therefore, the net positive charge on HHD2 would be enhanced at lower pH resulting in better binding between DNA and HHD2.

To probe the binding of HHD2 to DNA of different sequence and structures (ss, ds, and overhang DNAs), we designed following four DNA sequences, ssDNA-24, dsDNA-24, 5′ overhang DNA (5′ss_dsDNA-24), and 3′ overhang DNA (3′ss_dsDNA_24) (Supplementary Table S2). ITC experiments showed that ssDNA-24 interacts with HHD2 (Figure 7D) with a *K*_d_ of 1.32 ± 0.15 μM similar to ssDNA-12 and ssDNA-22 (Table 3). Double-stranded dsDNA-24 showed interaction with HHD2 (Figure 7E) with a *K*_d_ value of 1.95 ± 0.31 μM (Table 3). 5′ overhang containing 5′ss_dsDNA-24 (Figure 7F) and 3′ overhang containing 3′ss_dsDNA-24 (Figure 7G) showed interaction with HHD2 with *K*_d_ values of 2.58 ± 0.27 and 1.22 ± 0.09 μM, respectively (Table 3).

From all ITC titration results, we conclude that HHD2 binds to the single and double stranded DNA of varying sequence and size with the *K*_d_s in the range of ∼1–3 μM, with slightly higher affinity for ssDNA than the dsDNA. These results corroborate the NMR CSP derived observations on HHD2 interaction with ssDNA-22 and dsDNA-22 (Figure 6E and Figure S11E). Also, ITC results showed that HHD2 binds 3′ overhang DNA with slightly better affinity compared to the 5′ overhang DNA (Table 3). Altogether, these results unequivocally showed that HHD2 of RTEL1 interacts with DNA of different length, sequence, and forms (ss, ds, ss_ds junction DNA).

### RPA 32C and DNA compete for the same binding site on RTEL1 HHD2

We compared the HHD2–32C binding with the HHD2–DNA binding as observed in the NMR titration experiments (Table 1). About twenty-five times higher *K*_d_ value and approximately twenty times higher *K*_off_ rate were observed for HHD2–32C binding compared to the HHD2–ssDNA-22 binding. Similarly, about seventeen times higher *K*_d_ value and approximately eight times higher *K*_off_ rate were observed for HHD2–32C binding compared to the HHD2–dsDNA-22 binding. Taken together, the NMR titration results showed that HHD2 binds DNA with higher affinity than RPA 32C.

**Figure 8.**
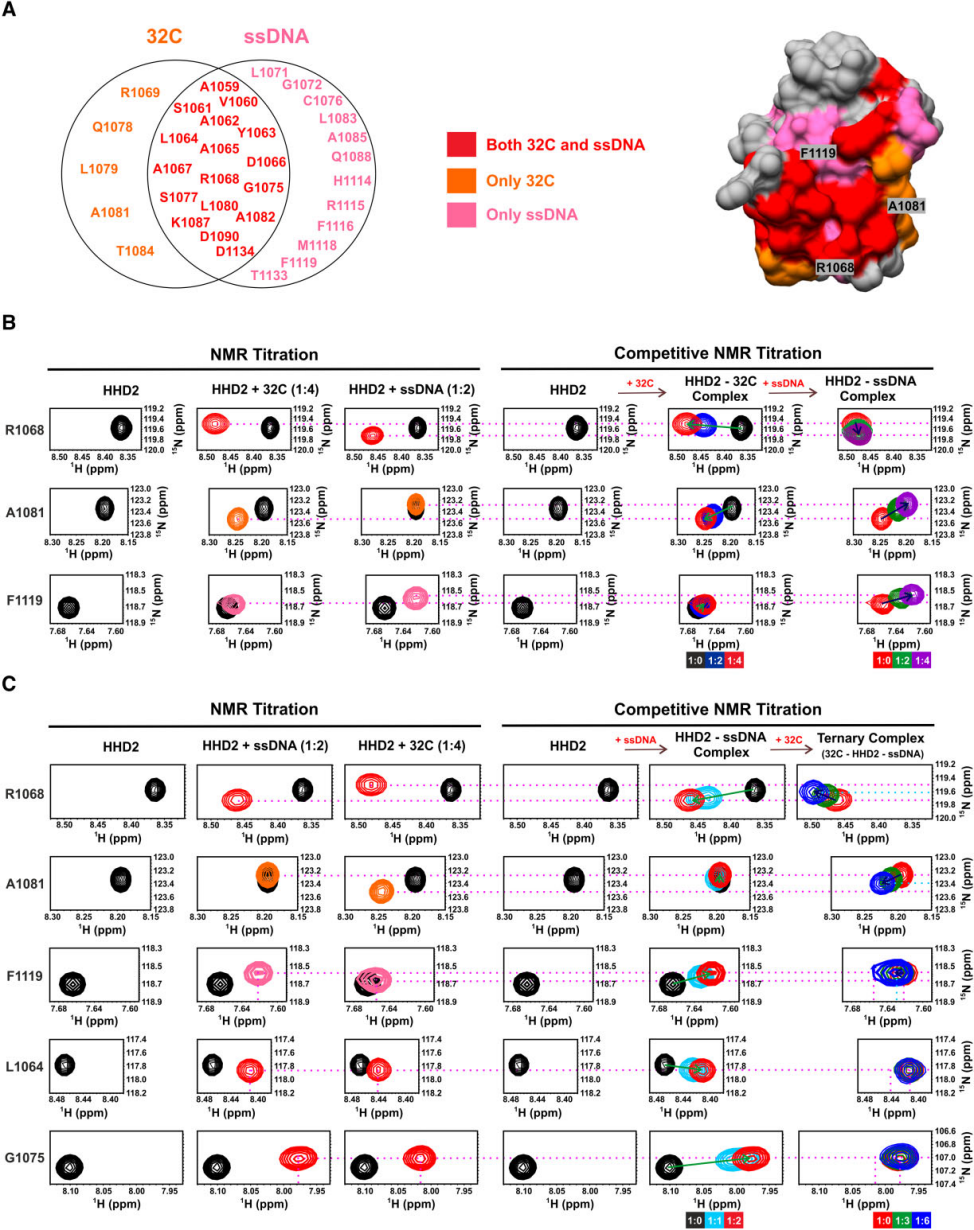
The interplay among RTEL1 HHD2, RPA 32C and DNA. (**A**) A subset of significantly perturbed residues of HHD2 upon binding to only RPA 32C (orange), only ssDNA (pink), and both 32C and ssDNA (red) are shown in Venn diagram. One representative residue from each subset is denoted on the surface-filled structure of HHD2. (**B**) ^1^H–^15^N HSQC cross-peaks trajectory of representative residues R1068, A1081, and F1119 of HHD2 upon titration with RPA 32C and ssDNA-22 at indicated molar ratios are shown (*left panel*). Competitive NMR titration (*right panel*) of ^15^N-labelled HHD2 with RPA 32C and ssDNA are shown. Arrow (green and blue) indicates the direction of movement of the cross-peaks upon titration. Initial and final peak positions are indicated by dashed line (pink). NMR titration of the HHD2–32C complex with DNA showed that the cross-peak of A1081 comes back to the free HHD2/HHD2–DNA complex position while the cross-peaks of F1119 and R1068 follow the path towards the HHD2-DNA complex. (**C**) ^1^H–^15^N HSQC cross-peaks trajectory of representative residues R1068, A1081, F1119, L1064 and G1075 of HHD2 upon titration with ssDNA-22 and RPA 32C at indicated molar ratios (*left panel*). Competitive NMR titration (*right panel*) of ^15^N-labelled HHD2 with ssDNA and RPA 32C are shown. Arrow (green and blue) indicates the direction of movement of the cross-peaks upon titration. Initial and final peak positions are indicated by dashed line (pink). Green dotted line indicates final position of cross-peaks in ternary complex. NMR titration of the HHD2–DNA complex with 32C showed that the cross-peak of A1081 and R1068 comes back only half-way to the HHD2-32C complex position while cross-peaks of L1064, and G1075 do not show interaction with 32C. The cross-peak of F1119 remains almost at the HHD2-DNA complex position even after addition of six molar equivalents of 32C.

The RPA 32C binding surface overlaps with the DNA binding surface in the HHD2 structure (Figure 8A). RPA 32C binds mainly to the solvent-exposed surfaces of helix H1 and H2, while DNA binds in the pocket formed by helix H1, H2, and H4, suggesting HHD2 adapts to bind DNA and 32C on the same surface. Overall, there are more number of HHD2 residues perturbed in case of ssDNA than 32C binding (Figure 8A), which may result in better affinity for HHD2-ssDNA complex than HHD2–32C complex (Table 1). Therefore, we hypothesized that RPA 32C and DNA may bind competitively to HHD2 of RTEL1. To test this hypothesis, we performed competitive binding experiments as described next.

We recorded a ^1^H–^15^N HSQC spectrum of ^15^N-labelled HHD2 bound to unlabelled 32C. The HHD2–32C complex was then titrated with the ssDNA-22. We visualised this competitive binding by monitoring the resonance cross peaks of three representative residues: R1068 (perturbed upon binding to both 32C and DNA), A1081 (perturbed only upon binding to 32C), and F1119 (perturbed only upon binding to DNA) (Figure 8B). Addition of four molar equivalents of ssDNA-22 to HHD2–32C complex resulted in R1068 cross peak from HHD2–32C complex shifting to the position corresponding to HHD2–ssDNA complex (Figure 8B). The resonance cross peak corresponding to A1081 from HHD2–32C complex shifts back to the position of free HHD2 upon addition of ssDNA (Figure 8B). The resonance cross peak of F1119 that does not show perturbation in HHD2–32C complex, shifts to the position corresponding to HHD2–DNA complex (Figure 8B). These results elegantly showed the competitive displacement of bound 32C from HHD2 by ssDNA-22.

We also probed the competitive binding of 32C and DNA for HHD2 by monitoring the ^1^H–^15^N HSQC spectra of ^15^N-labelled 32C (Figure S14). Four resonance cross peaks corresponding to residues E252, Y256, T258 and D268 were chosen to explain this. All of these residues of RPA 32C show chemical shift perturbations upon binding to HHD2 (Figure S14). Addition of four molar equivalents of ssDNA-22 to the 32C–HHD2 complex, resulted in complete reversal of chemical shifts of these residues to the position of free 32C (Figure S14). This reaffirmed the competitive displacement of bound 32C from HHD2 by ssDNA-22.

We then posed the question, whether 32C can displace the bound ssDNA from HHD2–ssDNA complex. This was probed by titrating the HHD2–ssDNA-22 complex by increasing concentration of 32C. Resonance peaks corresponding to residues R1068, A1081, F1119, L1064 and G1075 of HHD2 were selected to explain the results. The behaviour of R1068, A1081, F1119 upon interaction of HHD2 with 32C and DNA has been explained above. Residues L1064 and G1075 are perturbed upon binding to both 32C and DNA (similar to R1068) (Figure 8C).

Addition of 32C to HHD2–ssDNA-22 complex resulted in R1068 cross peak from HHD2–ssDNA-22 complex shifting towards the position corresponding to HHD2–32C complex. However, even after addition of six molar equivalents of 32C, R1068 peak does not shift completely to the position corresponding to HHD2–32C complex (Figure 8C). Cross peaks corresponding to L1064 and G1075 remain at the position corresponding to HHD2–ssDNA-22 complex and does not shift back to free HHD2 or HHD2–32C position (Figure 8C). Interestingly, the resonance cross peak corresponding to A1081 that does not show perturbation in HHD2–ssDNA-22 complex shifts the position corresponding to HHD2–32C complex upon addition of 32C (Figure 8C). This suggest that 32C is binding the HHD2–ssDNA complex resulting in a ternary complex of ssDNA–HHD2–32C. This result was further reinforced by observing residue F1119 that is perturbed only upon binding to ssDNA. Upon addition of 32C to the HHD2–ssDNA-22 complex, F1119 cross peak does not show any significant perturbation (Figure 8C).

In summary, the competitive titration results showed that RPA 32C is not able to compete out the bound ssDNA from HHD2 but rather forms a ternary complex of ssDNA–HHD2–32C. On the other hand, ssDNA is able to dislodge bound 32C from HHD2 resulting in HHD2–ssDNA complex formation. These results are in agreement with the titration results (ITC and NMR) that had shown that HHD2 binds ssDNA with higher affinity than the 32C.

## Discussion

### RTEL1 performs DNA damage repair through RPA-mediated recruitment at D-loop

RPA is the first responder to ssDNA and acts as a hub protein to recruit multiple specific factors (helicases, translocases, nucleases, etc.) to regulate DNA replication, repair, and recombination processes (57,58). RTEL1 and RPA showed nuclear co-localization under DNA damage conditions. Co-immunoprecipitation experiments showed that RPA and RTEL1 physically interact. We observed that deletion of the HHD1+2 region from RTEL1 significantly reduced RPA–RTEL1 interaction albeit not completely (Figure 1E). Since both RPA and RTEL1 are multi-domain proteins, we postulate that there might be other RPA interaction sites besides the HHD2 on RTEL1.

RPA interacts with its protein partners in a multivalent manner that results in high affinity binding, however the system remains dynamic and can be rapidly remodelled in the cell (59–61). Also, the interaction that involves individual domains of RPA and the partner proteins, although specific, is often weak. Several RPA interacting proteins like RAD52, XPA, SV40 Tag, WRN etc., show multivalent binding with RPA; primary interaction through RPA 70N or RPA 32C and secondary interaction within the tandem RPA 70A & 70B domains (58,59,61). Using extensive NMR titration-based experiments, we probed the binding of HHD1 and HHD2 of RTEL1 with different domains of RPA. The results showed that the RTEL1 HHD2 interacts primarily with the RPA 32C domain (Figure S5A–D, Figure S6A-C, and Figure 4A–D).

RPA binds ssDNA with high affinity (*K*_d_ in nM range) owing to the presence of multiple DNA binding OB-fold domains (DBDs) (20,60). The association and dissociation of individual DBDs lead to conformational dynamics of the ssDNA–RPA complex. The RPA interacting proteins selectively modulate the conformational dynamics of individual DBDs (62,63). Thus, RPA can pass the ssDNA to the interacting partner that has lower binding affinity for DNA (e.g. ∼ 1–3 μM *K*_d_ in case of RTEL1 HHD2–DNA binding).

**Figure 9.**
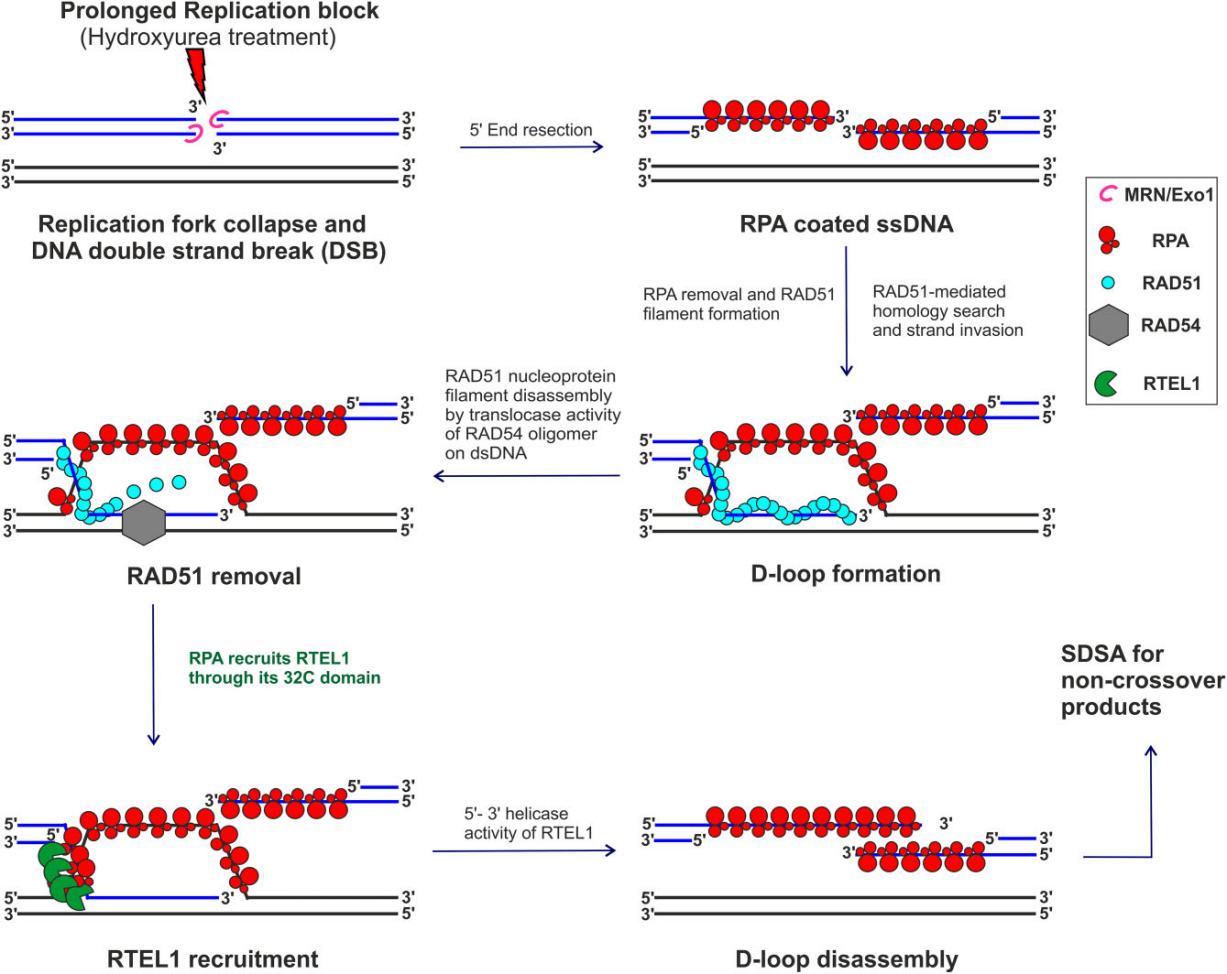
Proposed model of RPA mediated recruitment of RTEL1 at DNA repair sites. Prolonged replication block by hydroxyurea treatment eventually leads to DNA double-strand break (DSB). MRN/Exo1 (pink) complex executes 5′ end resection to generate the 3′ overhang, which quickly gets coated by RPA (red). RAD51 (cyan) nucleoprotein filament invades the homologous dsDNA to form D (displacement)-loop. RAD54 (grey) translocase activity helps in the removal of RAD51. RPA, present on the displaced ssDNA and invading strand, potentially will recruit the RTEL1 (green) on D-loop through its 32C domain. The 5′-3′ helicase activity of RTEL1 helps disassemble the D-loop. It thus directs the recombination intermediate to follow the synthesis-dependent strand-annealing (SDSA) pathway of homologous recombination to generate the non-crossover products.

Based on the existing literature and the results presented here, we refined the existing models describing the role of RPA in the recruitment of RTEL1 at the D-loop (Figure 9). RPA is present on the displaced ssDNA of the D-loop structure at DNA repair sites (64,65). Once the RAD54, HELQ-1 and RFS-1 displace the RAD51 from the invading strand of the D-loop (66–68), transiently exposed ssDNA of the invading strand gets occluded by RPA. The RPA, present on the invading strand and the displaced strand of the D-loop, is ready to recruit specific downstream factors. RPA 32C and RTEL1 HHD2 interaction would recruit RTEL1 at the ss-dsDNA junction of the invading strand of the D-loop. A conformational change in RPA (62) upon interaction with RTEL1 will expose the ssDNA, making it available for RTEL1 binding. It is noteworthy that the RTEL1 HHD2 domain has a higher affinity for ssDNA compared to its affinity for the RPA 32C (Table 1), which will facilitate the RPA-mediated recruitment of RTEL1 at the D-loop. Upon recruitment, RTEL1 would unwind the DNA at ss-dsDNA junction using its 5′-3′ DNA helicase activity and thus help resolve the D-loop structure (Figure 9).

In an *in vitro* assay, RTEL1 was shown to dissociate the D-loops with 3′ invasion preferentially (25). Interestingly, the efficient unwinding required the presence of the RPA (25). Our results and the proposed model of RPA-mediated recruitment of the RTEL1 helicase at the D-loop structure could explain the mechanism behind this observation.

### Distinct surface charge distribution imparts dual function to HHD2 of RTEL1

The X-ray crystal structure of the HHD2 domain of RTEL1 reported here is the first structure of any domains of RTEL1. The crystal structure of HHD2 determined in this study, NMR chemical shift indexing, and high-confidence alpha fold models show that HHD1 and HHD2 fold into a common globular bundle of five helices (Figure S15). However, the amino acids on the domain surfaces are poorly conserved, yielding distinct surface properties. The isoelectric point (pI) of HHD1 and HHD2 is 6.31 and 8.73, respectively. HHD1 has an acidic surface and a largely neutral opposite surface. However, HHD2 consists of distinct basic and acidic surfaces (Figure S15), suggesting that these two tandem domains may have distinct roles in RTEL1.

In plants and invertebrates, RTEL1 has only one HHD, while vertebrates have two tandem HHDs in their RTEL1 protein (9,14). The distinct surface properties and spatial separation through long unstructured linker regions may be advantageous for their interaction with different proteins to coordinate multiple cellular functions. For example, only HHD1 was shown to interact with SLX4 (17). A short alpha-helical C-terminal extension of HHD1 was found to be important for its interaction with SLX4, while the HHD2 has no such extension.

The HHD2 interacts with the 32C domain of RPA and DNA using an overlapping surface (helices H1 and H2). Interestingly, equivalent helices in the Harmonin and CCM2 HHDs were reported to constitute the protein-binding sites in them (14). Like several RPA interacting proteins, the HHD2 of RTEL1 interacts with the conserved binding surface of RPA 32C (Figure S7). Therefore, we conclude that the HHD2 of RTEL1 is a unique hub domain capable of mediating protein-protein and protein-DNA interactions.

### Future perspective on the interplay of RTEL1, RPA and DNA

The results presented here have suggested that HHD2 of RTEL1 interacts with the ssDNA with slightly better affinity and slower off-rates than the dsDNA (Table 1). HHD2 can bind the ss-dsDNA junction (5′ and 3′ overhang DNAs) as well. We have shown the competitive binding of RPA 32C and ssDNA for the HHD2 of RTEL1 (Figure 8B and C). This interplay of RPA–RTEL1–DNA interactions may help in understanding the mechanism of RTEL1 in the DNA replication, repair, and recombination processes. RTEL1 interacts with the PCNA through its PIP-box motif and helps genome-wide replication (8). RTEL1 facilitates bypass of the DNA-protein cross-links (DPCs) by replicative helicase CMG; interestingly, PIP box of the RTEL1 is not required for this function (69). This suggests additional mechanisms (apart from the PCNA-mediated) of RTEL1 recruitment at the replication fork. Future studies may unravel the possibility of RPA-mediated recruitment of the RTEL1 at the replication fork. It would be interesting to explore the effect of the abolishment of the RTEL1–RPA interaction on DNA repair, genome-wide DNA replication, DPC bypass, and telomere maintenance.

## Supplementary Material

gkad1208_Supplemental_File

## Data Availability

The atomic coordinates and structure factors for the RTEL1 HHD2 have been deposited in the Protein Data Bank under PDB accession code 7WU8.
