# Supplementary material for: Harmonin homology domain-mediated interaction of RTEL1 helicase with RPA and DNA provides insights into its recruitment to DNA repair sites
Source: Nucleic Acids Res. 2023 Dec 28;52(3):1450–70. doi: 10.1093/nar/gkad1208 (PMC10853778; doi:10.1093/nar/gkad1208)
Supplement: gkad1208_Supplemental_File [file gkad1208_supplemental_file.pdf]

SUPPLEMENTAL INFORMATION

Harmonin homology domain-mediated interaction of RTEL1 helicase with RPA and DNA provides insights into its recruitment to DNA repair sites

Niranjan Kumar[1], Arushi Taneja[2], Meenakshi Ghosh[1], Ulli Rothweiler[3], Nagalingam Ravi Sundaresan[2], and Mahavir Singh[1,*]

[1]Molecular Biophysics Unit, Indian Institute of Science, Bengaluru, 560012, India

[2]Department of Microbiology and Cell Biology, Indian Institute of Science, Bengaluru, 560012, India

[3]The Norwegian Structural Biology Centre, Department of Chemistry, The Arctic University of Norway, N-9037 Tromsø, Norway

Correspondence

Tel: +91 80 2293 2839; singh@iisc.ac.in (M.S.)

SUPPLEMENTARY FIGURES

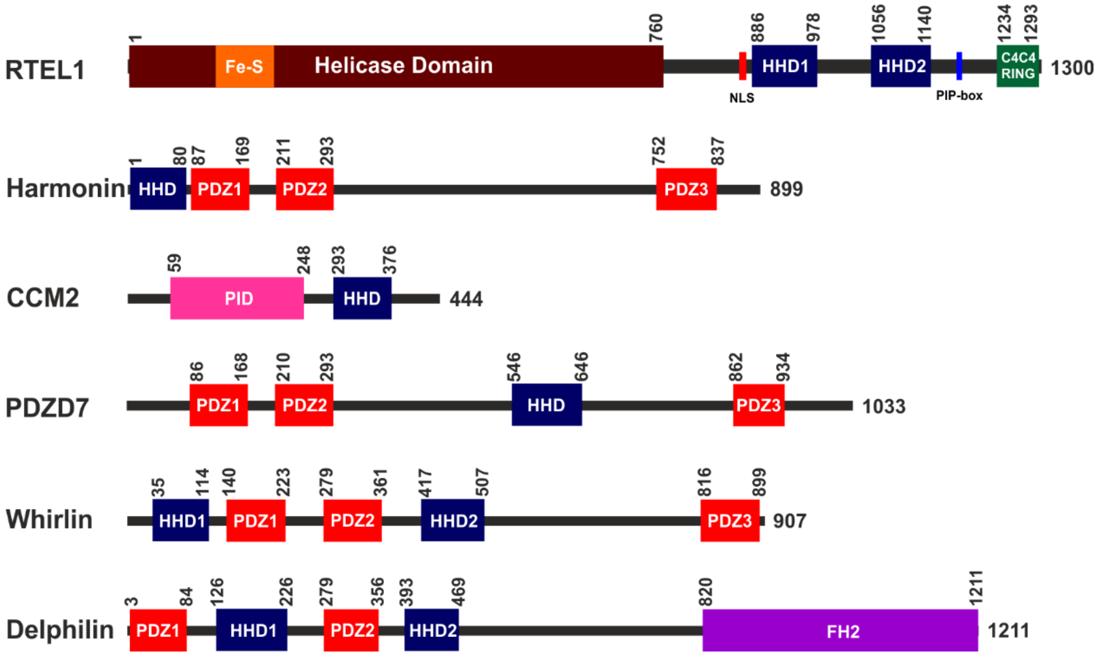

Figure S1. Schematic of domain organization of harmonin homology domains (HHDs) containing human proteins: RTEL1, Harmonin, CCM2, PDZD7, Whirlin, and Delphilin (10-13). Protein length and domain boundaries are mentioned. The HHDs are depicted as blue rectangles.

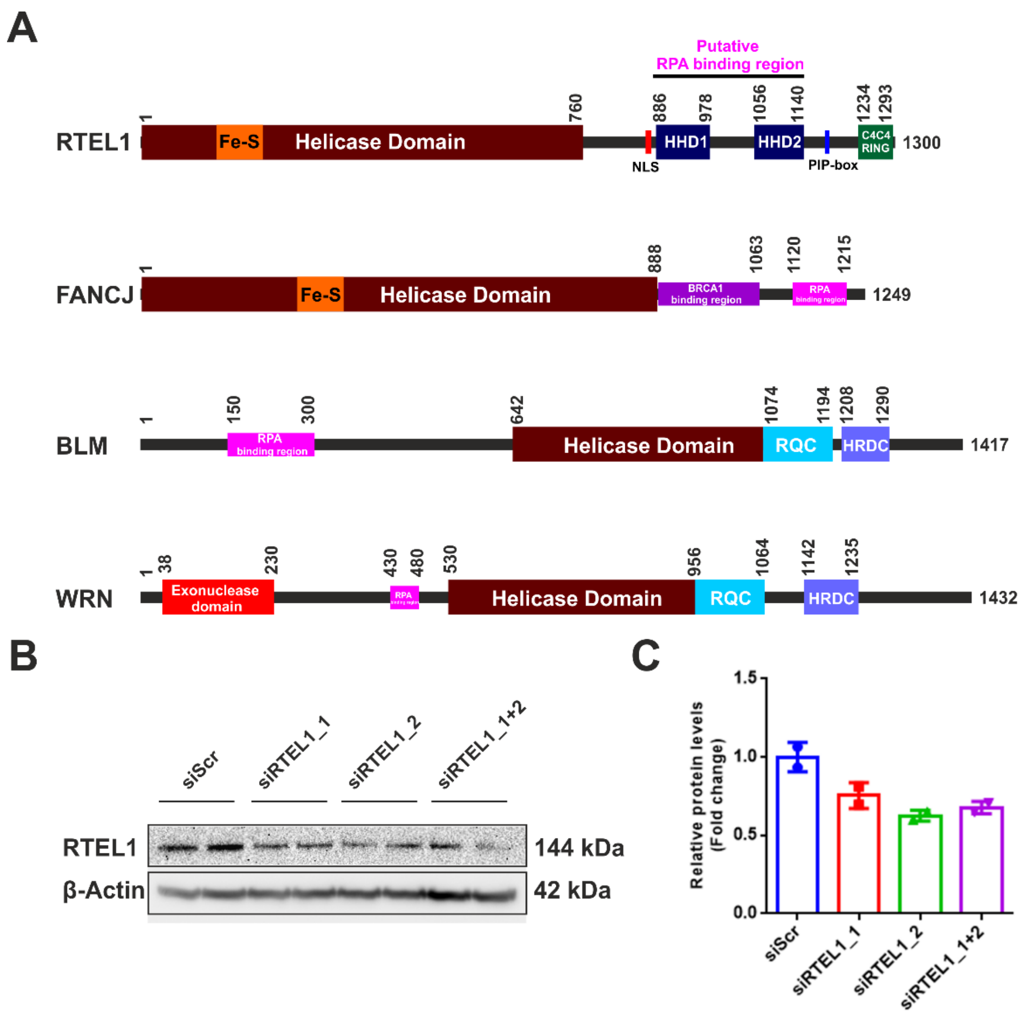

Figure S2. (A) Schematic of the domain organization of human RTEL1, FANCJ, BLM, and WRN helicases. The putative RPA binding region in RTEL1 is marked along with the known RPA binding region (pink rectangular box) in FANCJ, BLM, and WRN helicases (1,2). **(B)** Representative immunoblot showing knockdown of RTEL1. Whole cell extracts of U2OS cells transfected with siRNA against endogenous RTEL1 were analyzed through immunoblotting (IB) with indicated antibodies. **(C)** Bar graph showing relative protein level of endogenous RTEL1 in presence of indicated siRNA.

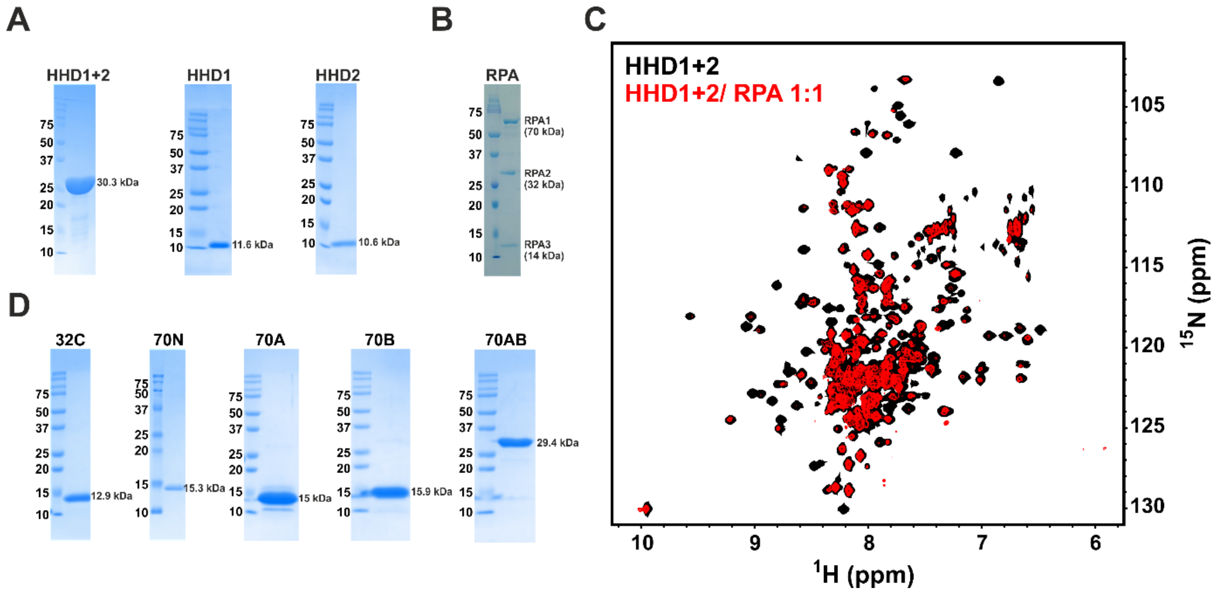

Figure S3. (A) SDS-PAGE gels of purified HHD1+2, HHD1, and HHD2. **(B)** SDS-PAGE gel of purified heterotrimeric complex of RPA. **(C)** Overlay of ^{1}H-^{15}N TROSY HSQC spectra of ^{15}N-labelled HHD1+2 in the absence (black) and presence (red) of RPA complex (at 1:1 molar ratio). **(D)** SDS-PAGE gels of purified 32C, 70N, 70A, 70B, and 70AB domains of RPA.

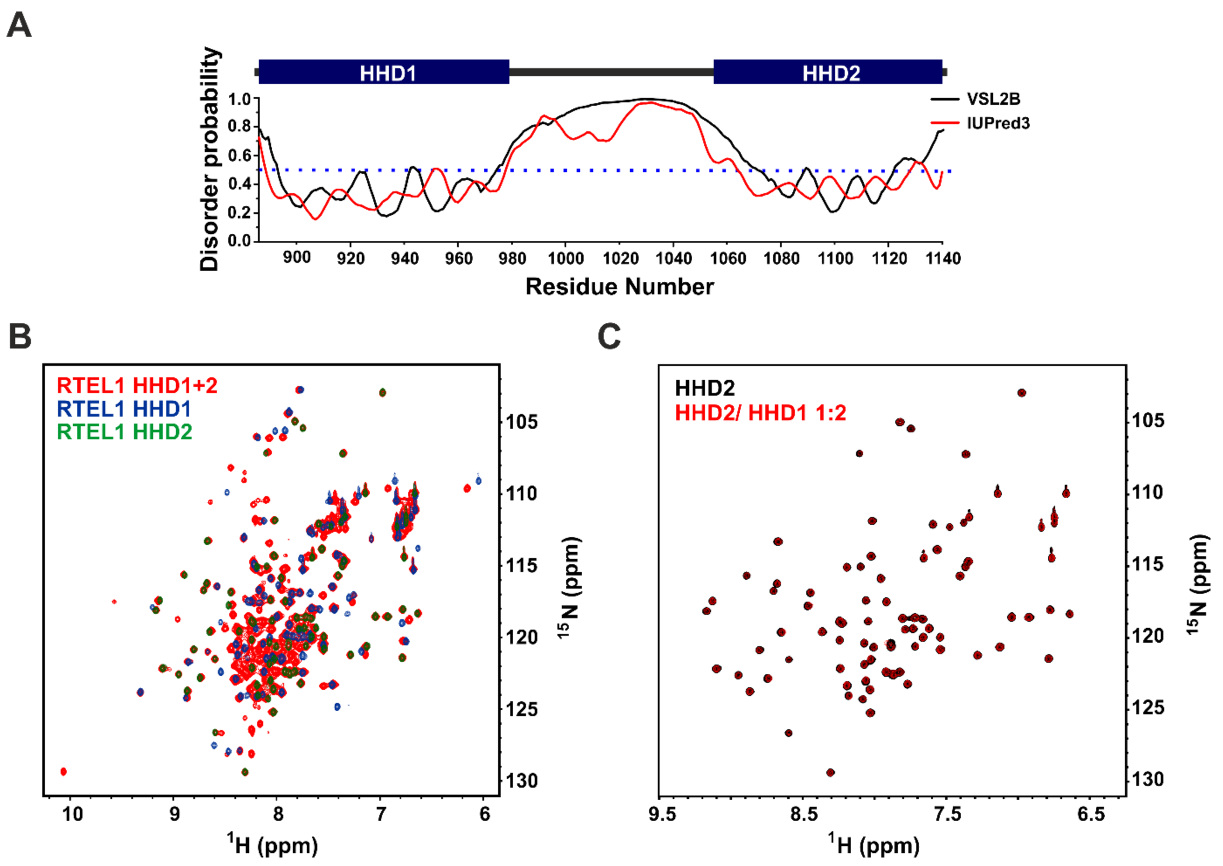

Figure S4. (A) Prediction of the intrinsically disordered region in the RTEL1 HHD1+2 domain using bioinformatic tools VSL2B (3) and IUPred3 (4). The blue dash line indicates the cut-off value (0.5) for disorder probability**. (B)** Overlay of ^{1}H-^{15}N HSQC spectra of HHD1 (blue), HHD2 (green), and HHD1+2 (red). **(C)** Overlay of ^{1}H-^{15}N HSQC spectra of ^{15}N-labelled HHD2 in the absence (black) and presence (red) of HHD1 (at 1:2 molar ratio). No significant CSPs were observed.

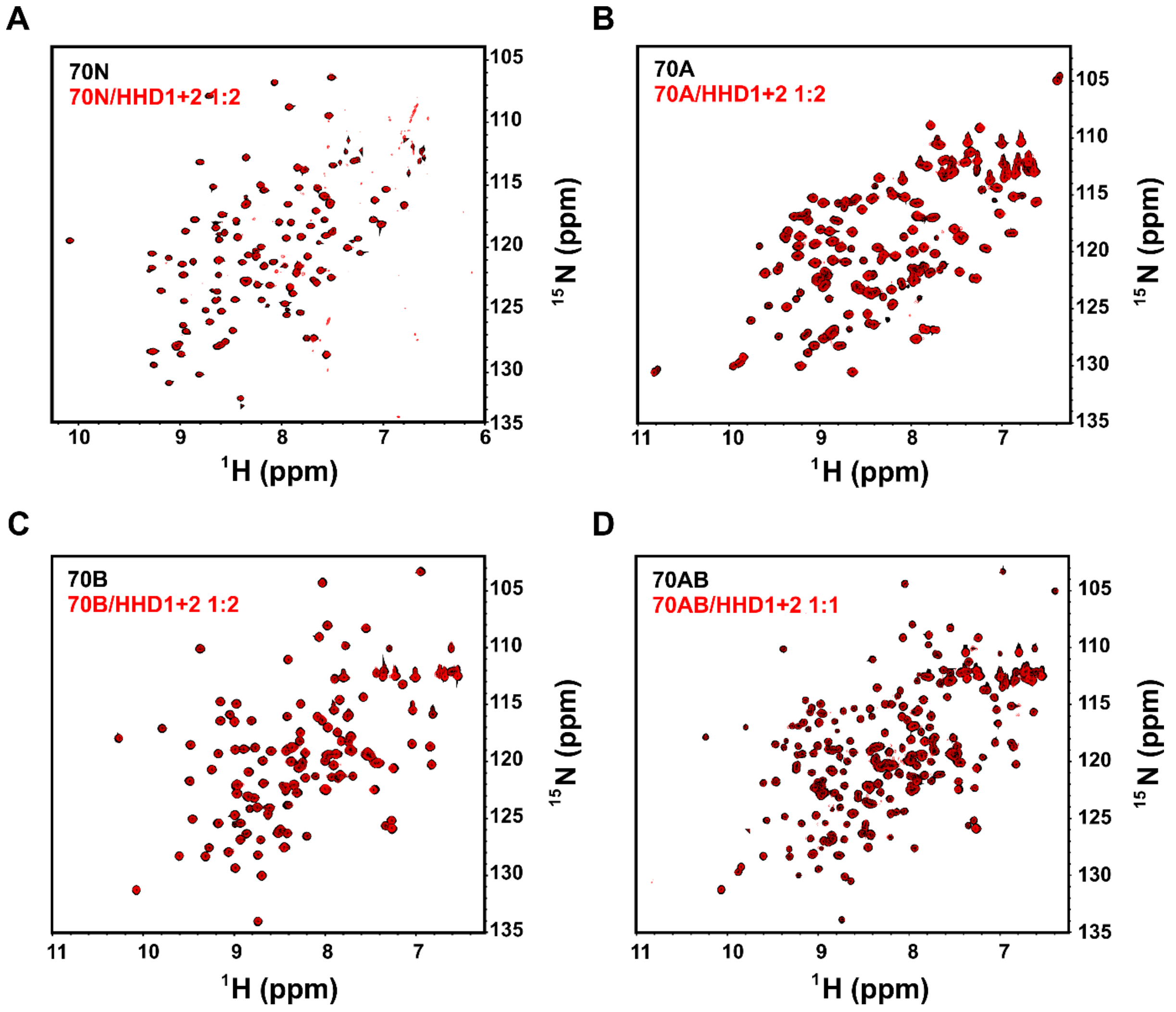

Figure S5. (A) Overlay of ^{1}H-^{15}N TROSY HSQC spectra of ^{15}N-labelled 70N in the absence (black) and presence (red) of HHD1+2 (at 1:2 molar ratio). **(B)** Overlay of ^{1}H -^{15}N HSQC spectra of ^{15}N-labelled 70A in the absence (black) and presence (red) of HHD1+2 (at 1:2 molar ratio). **(C)** Overlay of ^{1}H -^{15}N HSQC spectra of ^{15}N-labelled 70B in the absence (black) and presence (red) of HHD1+2 (at 1:2 molar ratio). **(D)** Overlay of ^{1}H -^{15}N HSQC spectra of ^{15}N-labelled 70AB in the absence (black) and presence (red) of HHD1+2 (at 1:1 molar ratio). No significant CSPs were observed in all the titrations.

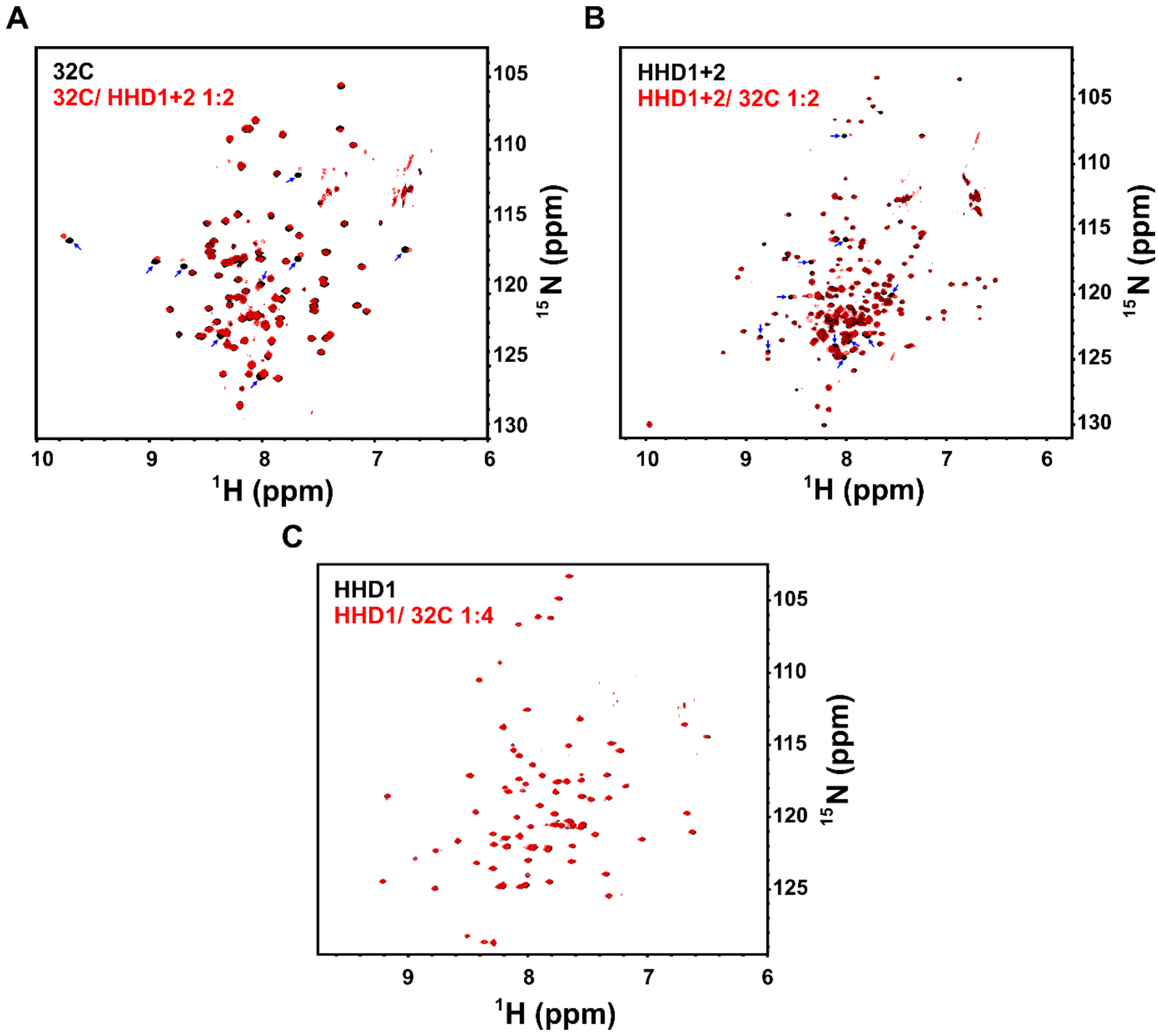

Figure S6. (A) Overlay of ^{1}H -^{15}N TROSY HSQC spectra of ^{15}N-labelled 32C in the absence (black) and presence (red) of HHD1+2 (at 1:2 molar ratio). Residues with large CSPs are marked (blue arrows). **(B)** Overlay of ^{1}H-^{15}N TROSY HSQC spectra of ^{15}N-labelled HHD1+2 in the absence (black) and presence (red) of 32C (at 1:2 molar ratio). Residues with large CSPs are marked (blue arrows). (**C)** Overlay of ^{1}H-^{15}N TROSY HSQC spectra of ^{15}N-labelled HHD1 in the absence (black) and presence (red) of 32C (at 1:4 molar ratio). No significant CSPs were observed in case of HHD1–32C titration.

Protein	Sequence of Interaction region	pI
RTEL1(1057-1089)	QHAVSAYLADARRALGSAGCSQLLAALTAYKQD	8.18
RAD52(246-278)	SSAVESEATHQRKLRQKQLQQQFRERMEKQQVR	10.88
ETAA1(886-918)	EEEKNRKCSPEEIQRKRQEALVRRMAKARASSV	10.01
XPA(16-48)	QPAELPASVRASIERKRQRALMLRQARLAARPY	11.88
UNG2(62-94)	PSSPLSAEQLDRIQRNKAAALLRLAARNVPVGF	11.54
SMARCAL1(1-33)	MSLPLTEEQRKKIEENRQKALARRAEKLLAEQH	9.52
TIPIN(191-223)	LSRSLTEEQQQRIERNKQLALERRQAKLLSNSQ	10.78
RPA32C (252-270)	EGHIYSTVDDDHFKSTDAE	4.22

Figure S7. RPA 32C-interacting region of different proteins involved in DNA repair and replication (5-9). The sequence corresponding to the interacting region of RPA 32C is depicted at the bottom. The theoretical pI values of the sequences indicate the net positive (blue) and net negative (red) charge, at physiological pH of these sequences.

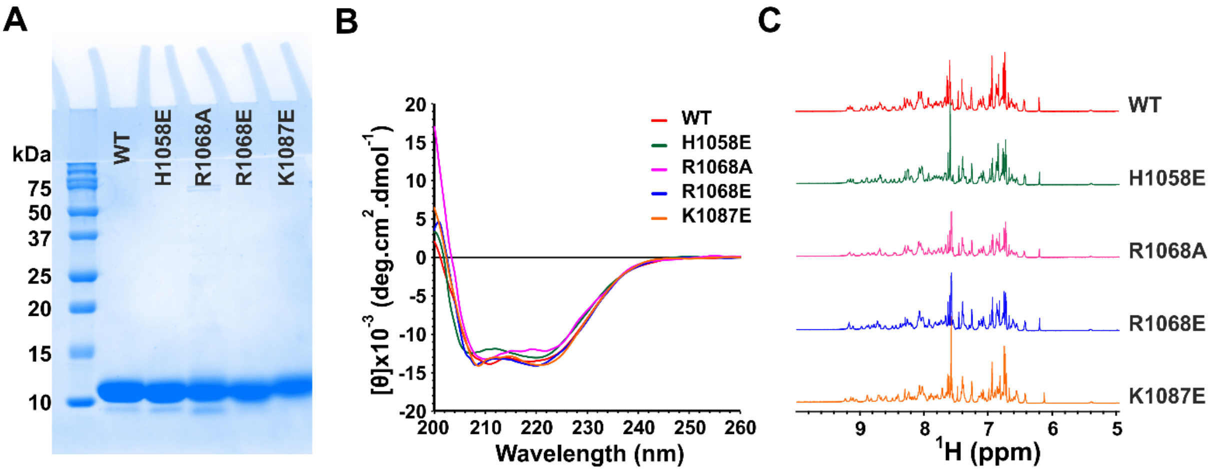

Figure S8. (A) SDS-PAGE gel of wild type (WT) and different mutants (H1058E, R1068A, R1068E, and K1087E) of HHD2. **(B)** CD spectra of WT HHD2 and mutants. **(C)** 1D ^{1}H NMR spectra (amino and aromatic protein region is shown) of WT HHD2 and mutants.

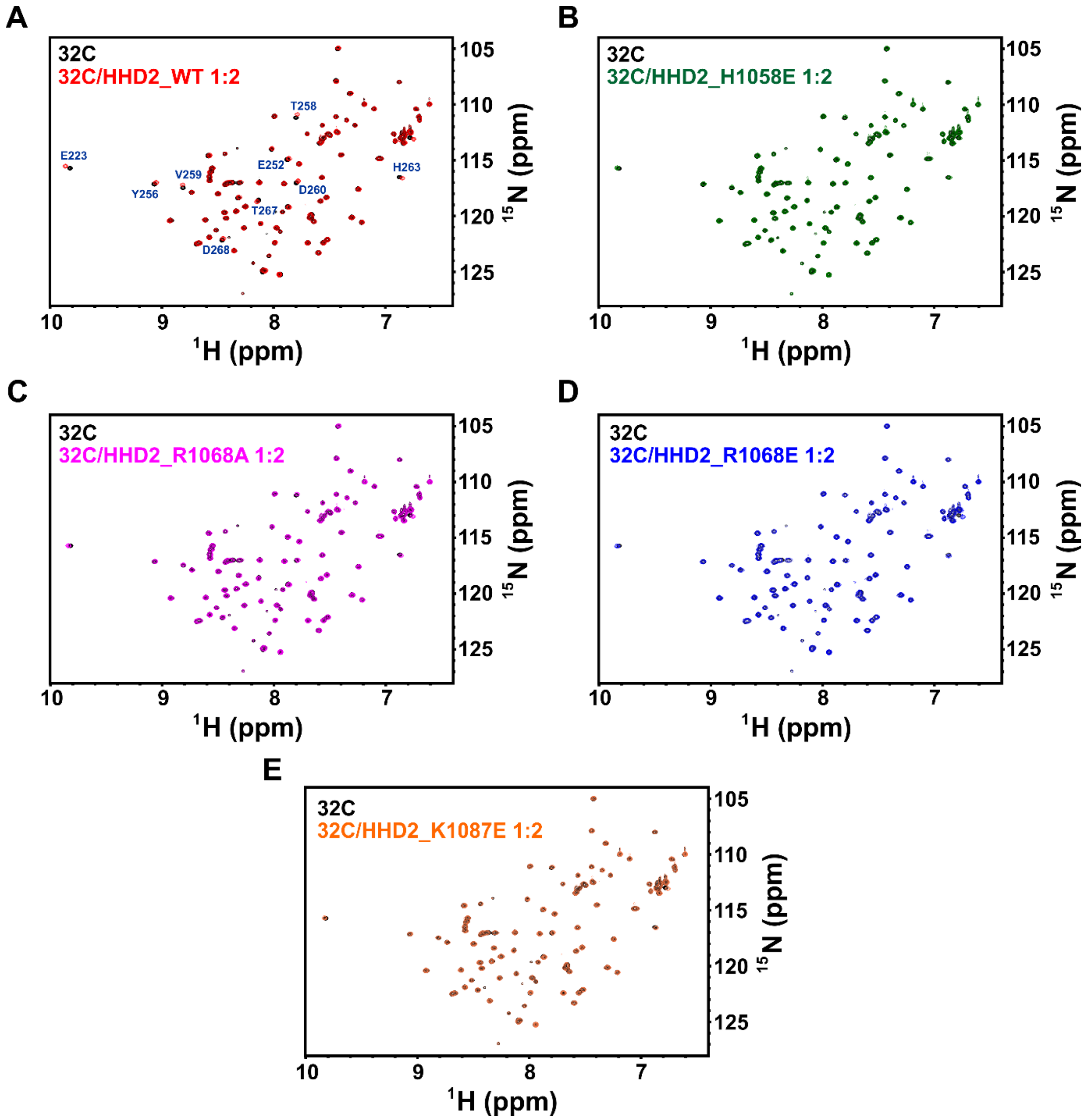

Figure S9. (A) Overlay of ^{1}H-^{15}N HSQC spectra of ^{15}N-labelled 32C in the absence (black) and presence (red) of wild type HHD2 (at 1:2 molar ratio). Residues with large CSPs are labelled (in blue). **(B)** Overlay of ^{1}H-^{15}N HSQC spectra of ^{15}N-labelled 32C in the absence (black) and presence (green) of H1058E mutant of HHD2 (at 1:2 molar ratio). **(C)** Overlay of ^{1}H-^{15}N HSQC spectra of ^{15}N-labelled 32C in the absence (black) and presence (magenta) of R1068A mutant of HHD2 (at 1:2 molar ratio). **(D)** Overlay of ^{1}H-^{15}N HSQC spectra of ^{15}N-labelled 32C in the absence (black) and presence (blue) of R1068E mutant of HHD2 (at 1:2 molar ratio). **(E)** Overlay of ^{1}H-^{15}N HSQC spectra of ^{15}N-labelled 32C in the absence (black)

and presence (orange) of K1087E mutant of HHD2 (at 1:2 molar ratio). The magnitudes of CSPs (observed in 32C spectra) were reduced in the mutant titration compared to WT HHD2 titration.

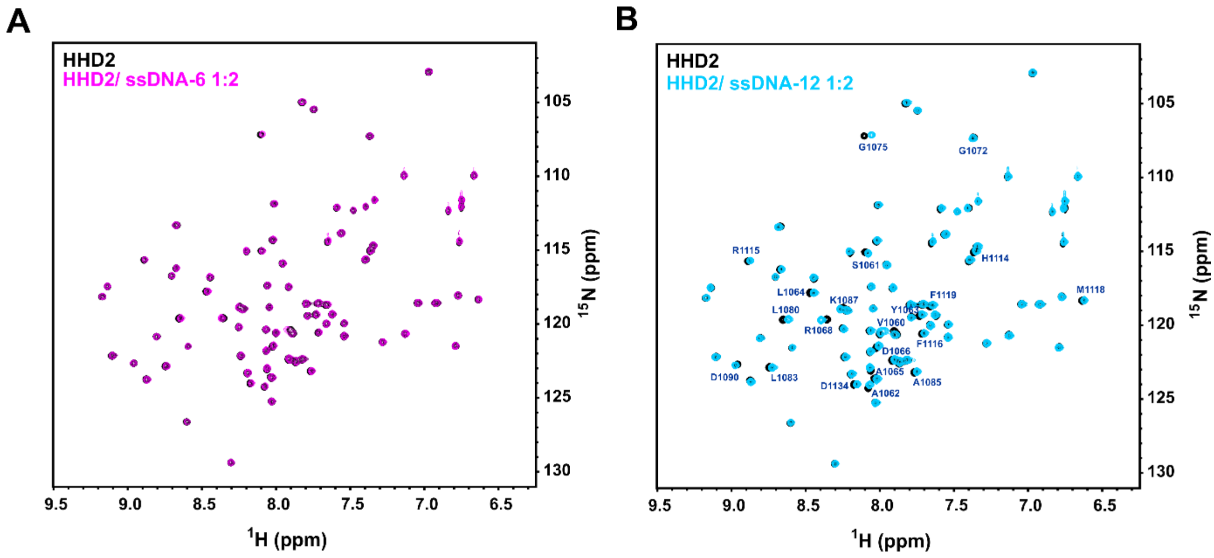

Figure S10. (A) Overlay of ^{1}H-^{15}N HSQC spectra of ^{15}N-labelled HHD2 in the absence (black) and presence (magenta) of ssDNA-6 (at 1:2 molar ratio). No significant CSPs were observed. **(B)** Overlay of ^{1}H-^{15}N HSQC spectra of ^{15}N-labelled HHD2 in the absence (black) and presence (sky blue) of ssDNA-12 (at 1:2 molar ratio). Residues with large CSPs are labelled (in blue).

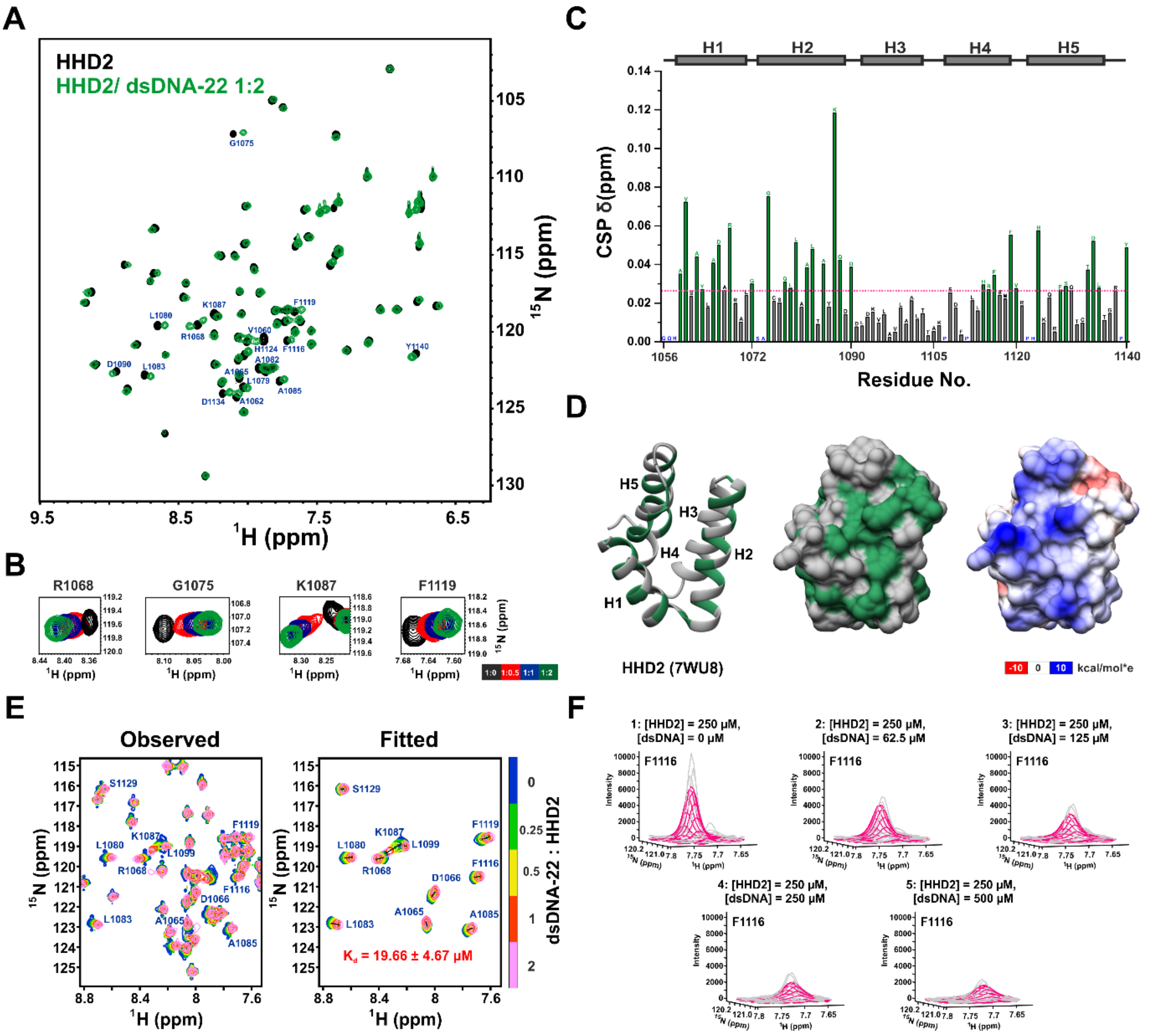

Figure S11. (A) Overlay of ^{1}H-^{15}N HSQC spectra of ^{15}N-labelled HHD2 in the absence (black) and presence (green) of dsDNA-22 (at 1:2 molar ratio). Residues with large CSPs are labelled (in blue). **(B)** ^{1}H-^{15}N cross-peaks trajectory of representative residues R1068, G1075, K1087, and F1119 of HHD2 upon titration with dsDNA-22 at indicated molar ratios. **(C)** Quantitation of CSPs in HHD2 upon titration with dsDNA-22 (at 1:2 molar ratio). Residues with more than average CSP (pink dash line) are marked as green bars and considered as significantly perturbed residues. Prolines and unassigned residues are colored in blue. The secondary structure corresponding to the HHD2 sequence is shown at the top. **(D)** Significantly perturbed residues (green) are marked on the structure of HHD2 (ribbon form on the *left* and surface form in the *middle*). Most of these residues lie on the positively charged surface (on the *right*) of HHD2. **(E)** 2D line shape analysis of the interaction of HHD2 with the dsDNA-22 using NMR

TITAN software (10). Observed and fitted spectral regions (zoomed in) are shown with indicated molar ratio of HHD2 and dsDNA-22 at each titration steps. 11 residues/spins (out of total 17 spins used for the analysis) along with the Fitted K_d is mentioned on the spectra. **(F)** Three-dimensional overlay of observed (gray) and fitted (magenta) peak of a representative spin F1116. Total concentration of HHD2 and dsDNA-22 are mentioned at each step of titration.

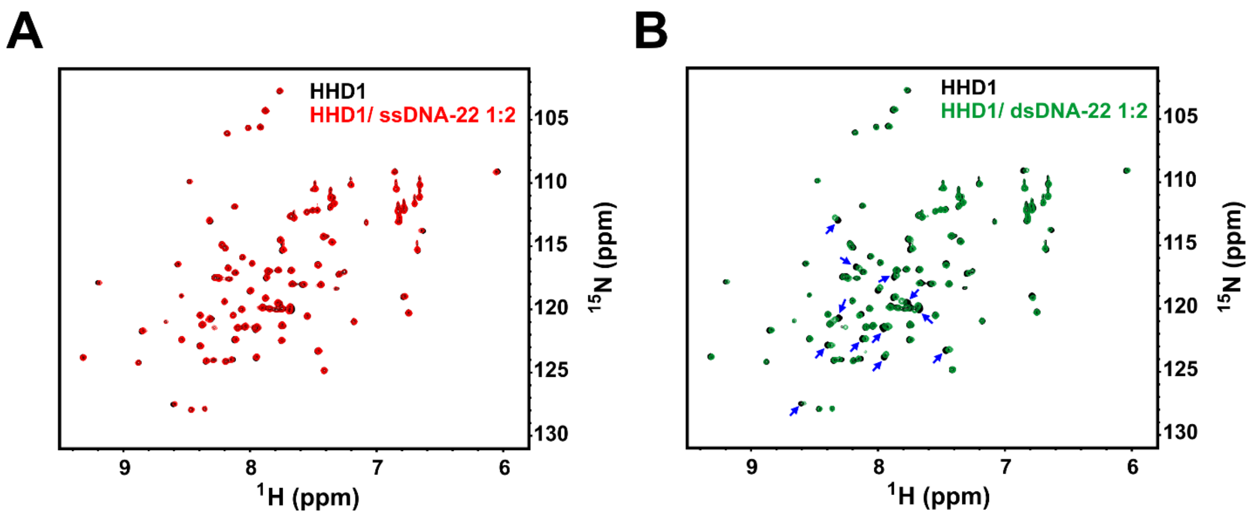

Figure S12. (A) Overlay of ^{1}H-^{15}N HSQC spectra of ^{15}N-labelled HHD1 in the absence (black) and presence (red) of ssDNA-22. No significant CSPs were observed. **(B)** Overlay of ^{1}H -^{15}N HSQC spectra of ^{15}N-labelled HHD1 in the absence (black) and presence (green) of dsDNA-22 (at 1:2 molar ratio). Residues that showed CSPs are marked (blue arrow).

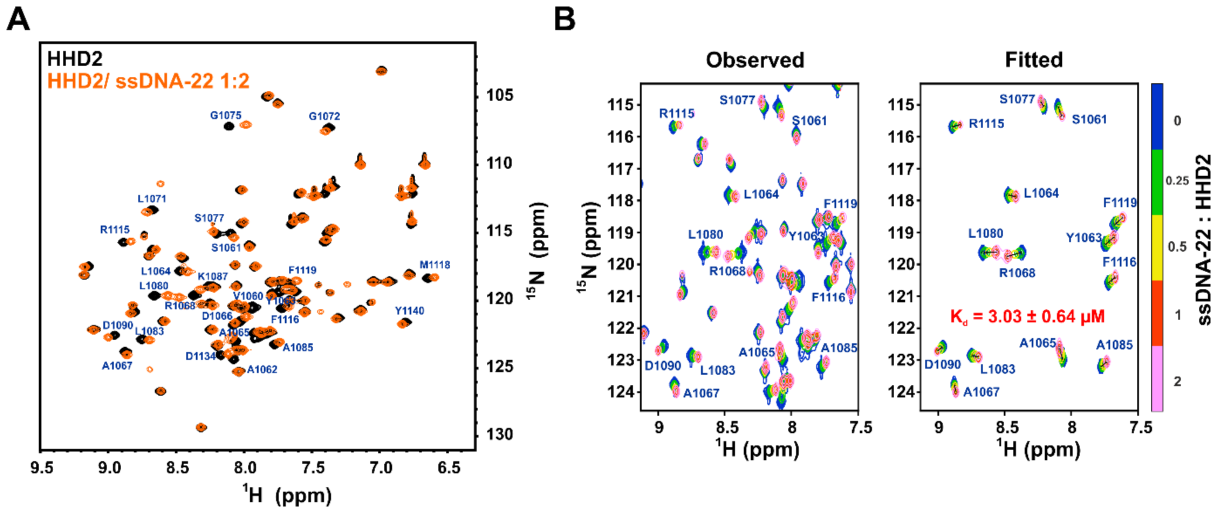

Figure S13. (A) Overlay of ^{1}H-^{15}N HSQC spectra of ^{15}N-labelled HHD2 in the absence (black) and presence (orange) of ssDNA-22 at pH 6.5. Residues that showed CSPs are marked (blue). **(B)** 2D line shape analysis of the interaction of HHD2 with the ssDNA-22 at pH 6.5 using NMR TITAN software (10). Observed and fitted spectral regions (zoomed in) are shown with indicated molar ratio of HHD2 and dsDNA-22 at each titration steps. 14 residues/spins (out of total 18 spins used for the analysis) along with the Fitted K_d is marked on the spectra.

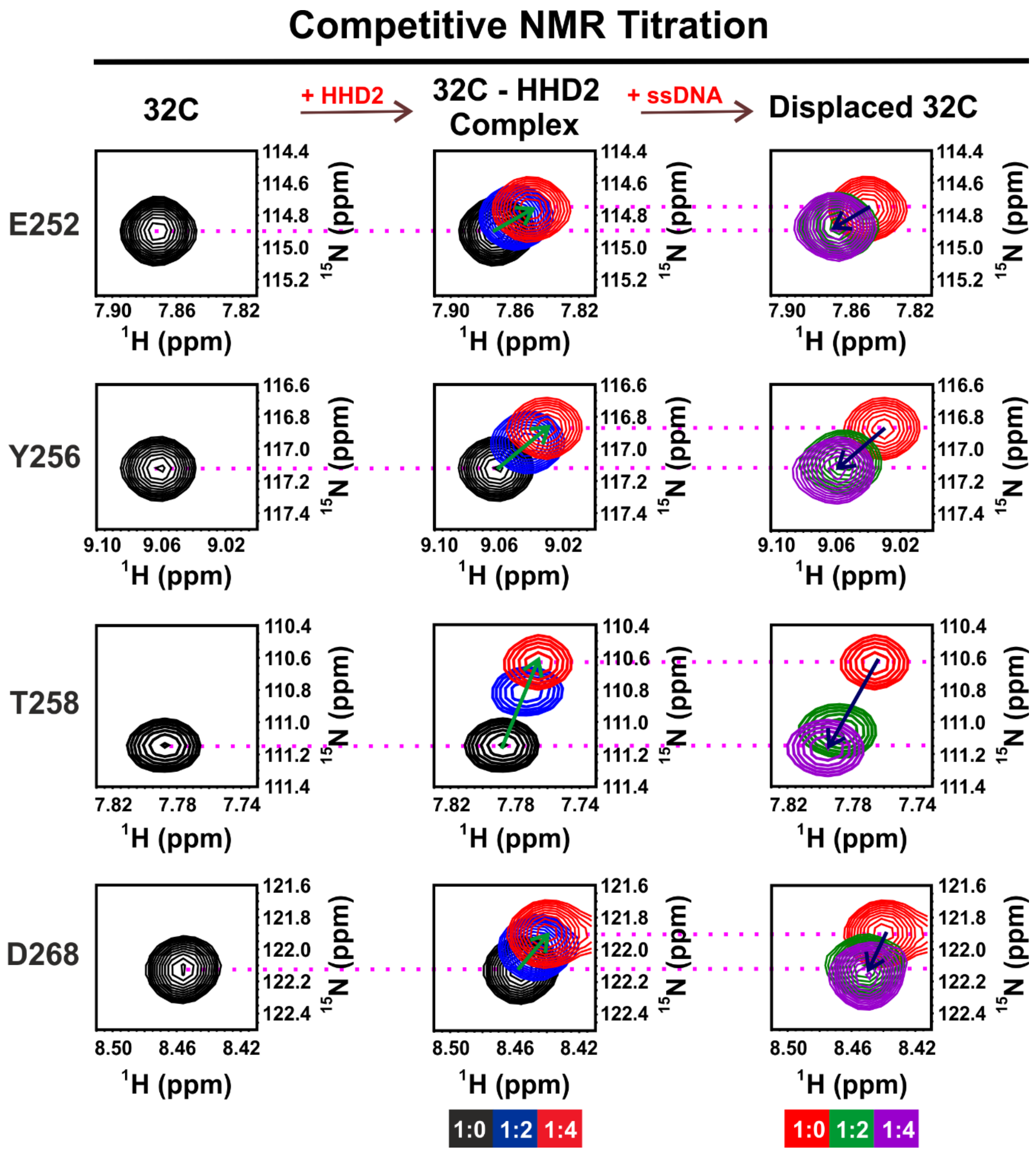

Figure S14. ^{1}H-^{15}N HSQC cross-peaks trajectory of representative residues E252, Y256, T258, and D268 of 32C upon competitive titration with HHD2 and ssDNA-22 at indicated molar ratios. Arrow (green and blue) indicates the direction of movement of the cross-peaks upon titration. Initial and final peak positions are indicated by dashed line (pink). NMR titration of the 32C–HHD2 complex with DNA showed that the cross-peaks of E252, Y256, T258, and D268 comes back to the free 32C position.

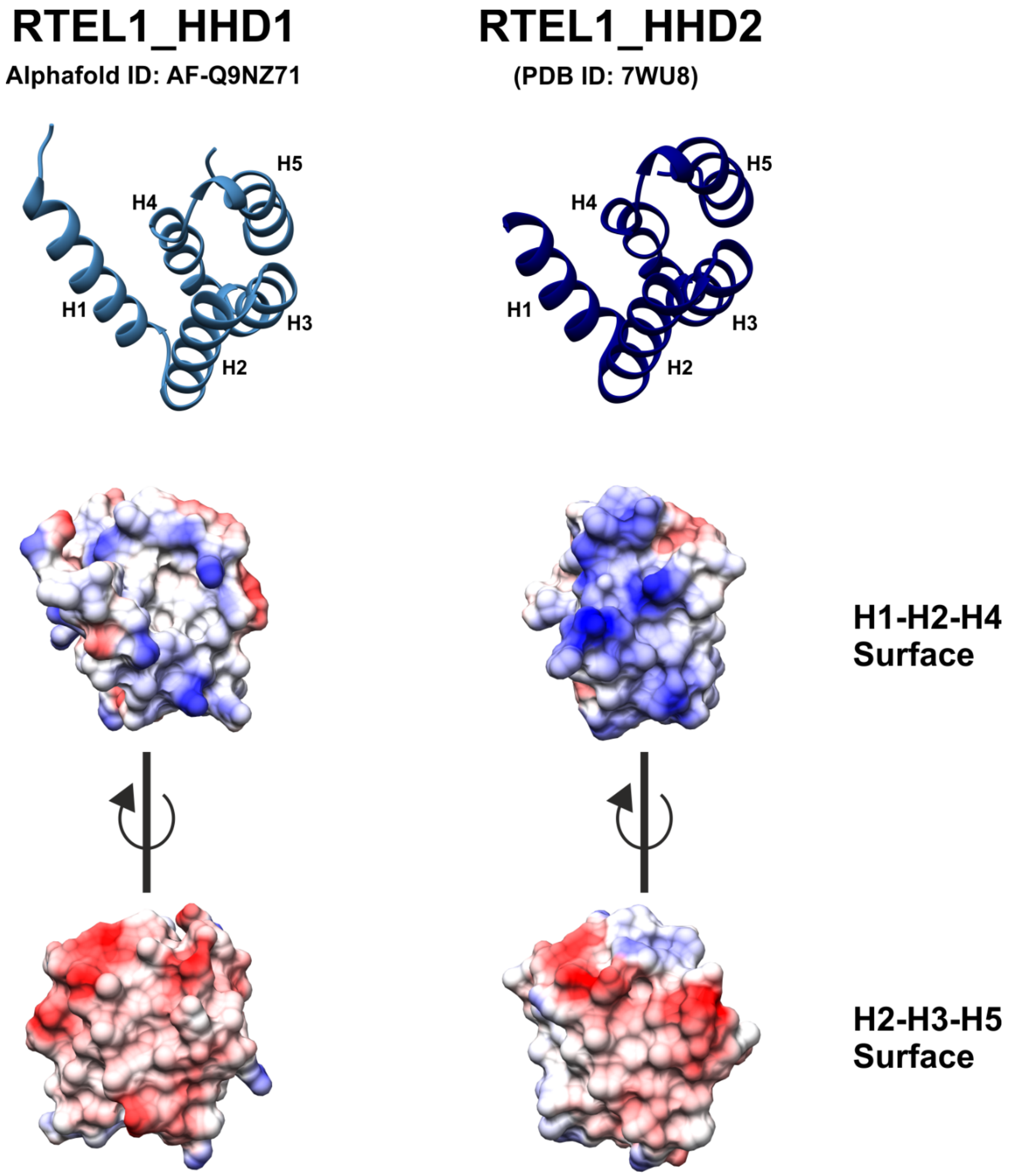

Figure S15. Comparison of alpha-fold model structure of HHD1 and crystal structure of HHD2 (reported in this study) (structure depicted in ribbon forms, *top panel*). Comparison of the surface electrostatic potential are shown *(bottom panel)*. HHD2 of RTEL1 shows a distinct positively charged surface (formed by helix H1, H2 and H4) and negatively charged surface (formed by helix H2, H3, and H5) in comparison to HHD1.

SUPPLEMENTARY TABLES

Table S1. Plasmids used in this study.

S. No.	Plasmids Name	Source	Identifier
1.	pcDNA3.1+N-HA-RTEL1	Genscript; This study	Clone ID OHu14298
2.	pcDNA3.1+N-HA-RTEL1-ΔHHD1+2	Genscript; This study	N/A
3.	p11d-tRPA (123)	Addgene (11)	Plasmid # 102613
4.	pET-28a (+) HHD1	(12)	N/A
5.	pET-28a (+) HHD2	(12)	N/A
6.	pET-28a (+) HHD1+2	This study	N/A
7.	pET-15b-RPA 70N	This study	N/A
8.	pET-15b-RPA 70A	This study	N/A
9.	pET-15b-RPA 70B	This study	N/A
10.	pET-15b-RPA 70AB	This study	N/A
11.	pET-15b-RPA 32C	This study	N/A
12.	pET-28a (+) HHD2_H1058E	This study	N/A
13.	pET-28a (+) HHD2_R1068A	This study	N/A
14.	pET-28a (+) HHD2_R1068E	This study	N/A
15.	pET-28a (+) HHD2_K1087E	This study	N/A

Table S2. Oligonucleotides used in this study.

S. No.	Oligonucleotides Name	Sequence (5'–3')
	Construction of plasmids for E. coli expression	
1.	HHD1+2_FP	GTCACATATGGAGCCCGTGG
2.	HHD1+2_RP	GTACCTCGAGCTAGTAGGGCCGG
3.	70N_FP	GTCACATATGGTCGGCCAGCTGAGCGAG
4.	70N_RP	GTCAGGATCCTTATTCATTATAGGGCACTGG
5.	70A_FP	GTCACATATGCAGTCCAAAGTGGTGCCCATTG
6.	70A_RP	GTCAGGATCCTTAGTCCTCACAGGGCATGACGG
7.	70B_FP	GTCACATATGCAGTTTGATTTCACGGGGATTG
8.	70B_RP	GTCAGGATCCTTATAAGGCTTGTCCTTCTGCG
9.	32C_FP	GTCACATATGGCCAACAGCCAGCCCTCA
10.	32C_RP	GTCAGGATCCTTATTCTGCATCTGTGGATTT
11.	HHD2_H1058E_FP	GGCCAGGAGGCCGTGAGCGCCTACCTG
12.	HHD2_H1058E_RP	GCTCACGGCCTCCTGGCCGCTGTGATGATG
13.	HHD2_R1068A_FP	GATGCCGCGAGGGCCCTGGGG
14.	HHD2_R1068A_RP	GGCCCTCGCGGCATCAGCCAGG
15.	HHD2_R1068E_FP	TGATGCCGAGAGGGCCCTGGGGTCC
16.	HHD2_R1068E_RP	CCCAGGGCCCTCTCGGCATCAGCCAGGTAGGC
17.	HHD2_K1087E_FP	ACAGCCTATGAGCAAGACGACGACCTCGACAAG
18.	HHD2_K1087E_RP	GTCGTCTTGCTCATAGGCTGTCAGCGCTGC
	DNA sample for ITC and NMR titrations	
19.	ssDNA-6	5'-TTAGGA-3'
20.	ssDNA-12	5'-TTAGGATTAGGA-3'
23.	ssDNA-22	5'-AGGATTAGGATTAGGATTAGGA-3'
24.	dsDNA-22	5'-AGGATTAGGATTAGGATTAGGA-3'<br>3'-TCCTAATCCTAATCCTAATCCT-5'
25.	ssDNA-24	5'-TTAGGCAGAGAGAGAGAGTTAGGC-3'
26.	dsDNA-24	5'-TTAGGCAGAGAGAGAGAGTTAGGC-3'<br>3'-AATCCGTCTCTCTCTCTCAATCCG-5'
27.	5'ss_dsDNA-24	5'-TTAGGCAGAGAGAGAGAGTTAGGC-3'<br>3'-TCTCTCTCTCTCAATCCG-5'
28.	3'ss_dsDNA-24	5'-TTAGGCAGAGAGAGAGAGTTAGGC-3'<br>3'-AATCCGTCTCTCTCTCTC-5'
	siRNA for RTEL1 knockdown	
29.	siRTEL1_1	5'-GGACAGGGCUCUCUAAUAA-3'
30.	siRTEL1_2	5'-GAGAAGCCCUGAGCUACCUUGGGGU-3'

Table S3. HADDOCK parameters of RTEL1 HHD2 – RPA 32C complex.

S. No.	Parameters	Cluster 1	Cluster 2	Cluster 3	Cluster 4	Cluster 5	Cluster 6	Cluster 7	Cluster 8	Cluster 9
1.	HADDOCK score	-78.6 ± 3.0	-54.5 ± 3.1	-60.3 ± 9.2	-57.9 ± 2.5	-67.3 ± 3.5	-32.7 ± 7.4	-42.1 ± 8.0	-47.7 ± 11.6	-51.1 ± 2.8
2.	Cluster size	79	22	13	12	11	4	4	4	4
3.	RMSD from the overall lowest-energy structure (Å)	0.7 ± 0.4	11.1 ± 1.3	2.4 ± 1.1	12.5 ± 0.1	5.1 ± 0.4	9.7 ± 0.6	3.7 ± 0.2	10.1 ± 0.6	4.0 ± 0.7
4.	Van der Waals energy (kcal mol^{-1})	-32.2 ± 3.5	-24.6 ± 4.5	-32.6 ± 6.8	-30.9 ± 1.5	-41.3 ± 4.0	-21.9 ± 4.8	-22.0 ± 5.1	-32.3 ± 4.2	-31.0 ± 3.1
5.	Electrostatic energy (kcal mol^{-1})	-317.4 ± 25.0	-221.2 ± 19.3	-205.8 ± 18.4	-188.8 ±17.8	-200.3 ± 14.3	-155.7 ± 31.7	-235.4 ± 19.8	-137.4 ± 51.8	-163.5 ± 22.9
6.	Desolvation energy (kcal mol^{-1})	6.9 ± 2.8	2.2 ± 1.9	2.6 ± 2.7	-0.5 ± 2.9	3.5 ± 2.3	2.9 ± 2.0	9.6 ± 3.1	1.5 ± 2.6	1.2 ± 3.9
7.	Restraints violation energy (kcal mol^{-1})	102.6 ± 25.3	120.9 ± 65.8	108.1 ±64.7	112.6 ±36.2	106.3 ± 38.4	173.8 ± 25.1	174.7 ± 26.5	105.3 ± 7.7	113.4 ± 16.5
8.	Buried Surface Area ($Å^2$)	1322.7 ± 29.4	1087.2 ± 36.7	1190.6 ± 80.1	1087.0 ± 86.4	1260.2 ± 34.8	910.5 ± 69.6	1026.2 ± 24.5	1029.9 ± 147.2	1009.6 ±66.8
9.	Z-score	-1.9	0.0	-0.4	-0.3	-1.0	1.7	1.0	0.5	0.3

SUPPLEMENTARY REFERENCES

1. Kang, D., Lee, S., Ryu, K.S., Cheong, H.K., Kim, E.H. and Park, C.J. (2018) Interaction of replication protein A with two acidic peptides from human Bloom syndrome protein. *FEBS Lett*, **592**, 547-558.
2. Yeom, G., Kim, J. and Park, C.J. (2019) Investigation of the core binding regions of human Werner syndrome and Fanconi anemia group J helicases on replication protein A. *Scientific reports*, **9**, 14016.
3. Obradovic, Z., Peng, K., Vucetic, S., Radivojac, P. and Dunker, A.K. (2005) Exploiting heterogeneous sequence properties improves prediction of protein disorder. *Proteins*, **61 Suppl 7**, 176-182.
4. Erdős, G., Pajkos, M. and Dosztányi, Z. (2021) IUPred3: prediction of protein disorder enhanced with unambiguous experimental annotation and visualization of evolutionary conservation. *Nucleic Acids Research*, **49**, W297-W303.
5. Mer, G., Bochkarev, A., Gupta, R., Bochkareva, E., Frappier, L., Ingles, C.J., Edwards, A.M. and Chazin, W.J. (2000) Structural basis for the recognition of DNA repair proteins UNG2, XPA, and RAD52 by replication factor RPA. *Cell*, **103**, 449-456.
6. Ali, S.I., Shin, J.S., Bae, S.H., Kim, B. and Choi, B.S. (2010) Replication protein A 32 interacts through a similar binding interface with TIPIN, XPA, and UNG2. *Int J Biochem Cell Biol*, **42**, 1210-1215.
7. Feldkamp, M.D., Mason, A.C., Eichman, B.F. and Chazin, W.J. (2014) Structural analysis of replication protein A recruitment of the DNA damage response protein SMARCAL1. *Biochemistry*, **53**, 3052-3061.
8. Xie, S., Lu, Y., Jakoncic, J., Sun, H., Xia, J. and Qian, C. (2014) Structure of RPA32 bound to the N-terminus of SMARCAL1 redefines the binding interface between RPA32 and its interacting proteins. *Febs j*, **281**, 3382-3396.
9. Bass, T.E., Luzwick, J.W., Kavanaugh, G., Carroll, C., Dungrawala, H., Glick, G.G., Feldkamp, M.D., Putney, R., Chazin, W.J. and Cortez, D. (2016) ETAA1 acts at stalled replication forks to maintain genome integrity. *Nat Cell Biol*, **18**, 1185-1195.
10. Waudby, C.A., Ramos, A., Cabrita, L.D. and Christodoulou, J. (2016) Two-Dimensional NMR Lineshape Analysis. *Scientific reports*, **6**, 24826.
11. Henricksen, L.A., Umbricht, C.B. and Wold, M.S. (1994) Recombinant replication protein A: expression, complex formation, and functional characterization. *J Biol Chem*, **269**, 11121-11132.
12. Kumar, N., Ghosh, M., Manikandan, P., Basak, S., Deepa, A. and Singh, M. (2022) Resonance assignment and secondary structure of the tandem harmonin homology domains of human RTEL1. *Biomol NMR Assign*, **16**, 159-164.
